# Placenta-specific1 (PLAC1) is a potential target for antibody-drug conjugate-based prostate cancer immunotherapy

**DOI:** 10.1038/s41598-017-13682-9

**Published:** 2017-10-17

**Authors:** Mohammad-Reza Nejadmoghaddam, Amir-Hassan Zarnani, Ramin Ghahremanzadeh, Roya Ghods, Jafar Mahmoudian, Maryam Yousefi, Mahboobeh Nazari, Mohammad Hossein Ghahremani, Maryam Abolhasani, Ali Anissian, Morteza Mahmoudi, Rassoul Dinarvand

**Affiliations:** 1 Nanotechnology Research Center, Faculty of Pharmacy, Tehran University of Medical Sciences (TUMS), Tehran, Iran; 2 Nanobiotechnology Research Center, Avicenna Research Institute, ACECR, Tehran, Iran; 3 Reproductive Immunology Research Center, Avicenna Research Institute, ACECR, Tehran, Iran; 4 Immunology Research Center, Iran University of Medical Sciences, IUMS, Tehran, Iran; 5 Oncopathology Research Center, Iran University of Medical Sciences, IUMS, Tehran, Iran; 6 Department of Molecular Medicine, Faculty of Advanced Technologies in Medicine, Iran University of Medical Sciences, IUMS, Tehran, Iran; 7 Monoclonal Antibody Research Center, Avicenna Research Institute, ACECR, Tehran, Iran; 8 Department of Pathology, Hasheminejad Kidney Center, Iran University of Medical Sciences, IUMS, Tehran, Iran; 9 Veterinary department, Islamic Azad University, Abhar branch, Abhar, Iran; 10 Department of Pharmaceutics, Faculty of Pharmacy, Tehran University of Medical Sciences, Tehran, Iran

**Keywords:** Cancer immunotherapy, Targeted therapies, Drug delivery, Prostate

## Abstract

Our recent findings strongly support the idea of PLAC1 being as a potential immunotherapeutic target in prostate cancer (PCa). Here, we have generated and evaluated an anti-placenta-specific1 (PLAC1)-based antibody drug conjugate (ADC) for targeted immunotherapy of PCa. Prostate cancer cells express considerable levels of PLAC1. The Anti-PLAC1 clone, 2H12C12, showed high reactivity with recombinant PLAC1 and selectivity recognized PLAC1 in prostate cancer cells but not in LS180 cells, the negative control. PLAC1 binding induced rapid internalization of the antibody within a few minutes which reached to about 50% after 15 min and almost completed within an hour. After SN38 conjugation to antibody, a drug-antibody ratio (DAR) of about 5.5 was achieved without apparent negative effect on antibody affinity to cell surface antigen. The ADC retained intrinsic antibody activity and showed enhanced and selective cytotoxicity with an IC50 of 62 nM which was about 15-fold lower compared to free drug. Anti-PLAC1-ADC induced apoptosis in human primary prostate cancer cells and prostate cell lines. No apparent cytotoxic effect was observed in *in vivo* animal safety experiments. Our newly developed anti-PLAC1-based ADCs might pave the way for a reliable, efficient, and novel immunotherapeutic modality for patients with PCa.

## Introduction

Prostate cancer (PCa) is the second leading cause of cancer death in men^1^. Based on limitations of currently available prostate cancer markers, there is a steadily growing interest for discovery of new biomarkers to accurately diagnosis and treat PCa. Despite numerous efforts and introduction of new PCa biomarkers (e.g., α_2_β_1_^hi^/CD133^+^/CD44^+^, STEAP1, and TENB2)^2–7^, the prognostic value of these markers in prostate tumors remains elusive^8,9^. Moreover, many normal cell types also express substantial levels of the predetermined markers reflecting ambiguity regarding their usefulness for practical immunotherapy targets.

Our recent findings on differential expression of placenta-specific 1 (PLAC1) in PCa and more importantly its positive association with Gleason score highlight the potential application of PLAC1 for targeted PCa therapy especially for patients with advanced stage of disease^8^. Moreover, we recently reported that PLAC1 is highly expressed by cancer cell lines of different histologically origins including those from breast, ovary, and prostate where we observed membrane-associated expression of this marker in some cancer cell lines including those originated from prostate cancer^10^. These findings were in line with other reports showing surface expression of PLAC1^11,12^ and strongly pointed to the potential usefulness of PLAC1 as a promising target for PCa immunotherapy^8,13^. We have recently produced a set of novel anti-human PLAC1 monoclonal antibodies (mAb) and have assessed their potential effectiveness in modulating different cancer-associated hallmarks including proliferation, invasion and resistance to apoptosis. We found that despite their high specific reactivity^10^, most of the anti-PLAC1 antibodies failed to exert substantial effects in the features mentioned above. To this end, we hypothesized that utilizing advantages of cancer cell specificity of anti-PLAC1 antibodies and cytotoxic activity of a chemically supertoxic agent could be considered an ideal approach for generation of an antibody drug conjugate (ADC) platform for targeted immunotherapy of PCa. This approach represents an innovative nanotherapeutics modality in which the antibody functions as self-targeting nanoscale carrier delivering a highly potent chemically cytotoxic agent^14,15^ into the antigen-expressing tumor cells; thereby triggering higher toxic effects than the free cytotoxic agent and increasing both the efficacy and safety of therapy^16^. In fact, clinical translation of antibody-mediated nanomedicine has been re-energized by generation of ADCs^17^ and they have become one of most dynamic drug classes, particularly in oncology. Recently, two FDA-approved ADCs (Adcetris^®^ and Kadcyla^®^) and ongoing clinical trials of more than 55 other ADC molecules have highlighted ADC development as an innovative nanotherapeutics approach for cancer treatment^18^.

To pursue potential application of anti-PLAC1 antibodies for cancer immunotherapy, we developed an anti-PLAC1-ADC and assessed its potential efficacy in three human prostate cancer cell lines, namely LNCaP, DU145, and PC3. Our data suggest that anti-PLAC1 ADC has a great potential as a therapeutic agent for tumors with PLAC1 overexpression.

## Results

### Generation of anti-PLAC1 antibodies

**Figure 1:**
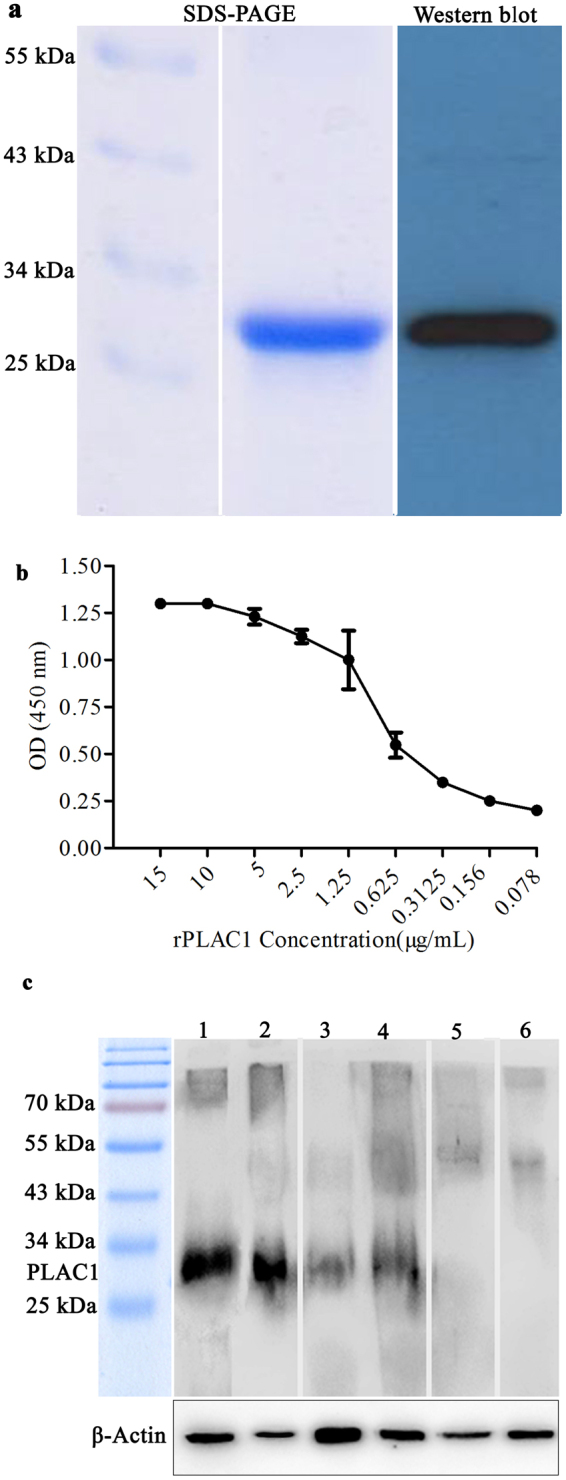
Characterization of recombinant PLAC1 and anti-PLAC1 antibodies. rhPLAC1was produced in *E. coli* and purified. The purity of the protein was assessed by SDS-PAGE analysis and Western blot using anti-His tag antibody (**a**) (full length gel and blot in supplementary information file, Fig. S6a,b). Monoclonal anti-PLAC1 antibody was generated in mice using rhPLAC1 as immunogen and reactivity of the purified anti-PLAC1 mAb, 2H12C12, was confirmed by indirect ELISA using rhPLAC1 as coating layer. Data were generated from at least three independent expeiments (**b**). Anti-PLAC1 mAb failed to react with PLAC1 in Western blotting and hence rabbit polyclonal anti-PLAC1 antibody was produced by immunizing the animals against rhPLAC1 and its reactivity with human placental PLAC1 was confirmed by Western blotting. In all lanes, lysate of human placenta has been loaded. Lanes 1, 2: Probed with purified rabbit anti-PLAC1 antibody. Lanes 3 and 4: Probed with PLAC1 hyperimmune rabbit serum. Lane 5: Probed with anti-PLAC1 mAb, 2H12C12. Lane 6: Probed with pre-immune rabbit IgG. Beta actin was used as loading control (**c**). After SDS-PAGE and transfer of proteins to the membrane, it was cut and different lanes were probed separately with different antibodies.

For generation of polyclonal and monoclonal anti-PLAC1 antibodies, rhPLAC1 was produced in a prokaryotic expression system. The rhPLAC1 was expressed as inclusion bodies and solubilization was done with buffer containing 2 M Urea. The purified proteins had more than 95% purity as detected by SDS-PAGE analysis. Western blot analysis demonstrated a sharp band of ~27 kDa corresponding to the estimated molecular weight of the recombinant protein after on-column refolding (Fig. 1a). This protein was then used as immunogen for generation of anti-PLAC1 antibodies. Monoclonal anti-PLAC1 antibody, 2H12C12, showed an excellent reactivity with the immunizing rhPLAC1 in ELISA yielding optical density of about one with coating concentration of 1.25 µg/mL (Fig. 1b). However, this mAb failed to work in Western blotting. In this regard, a rabbit polyclonal antibody was produced using the same immunogen and the results of preliminary experiments clearly showed reactivity of this antibody with human placental PLAC1 in Western blot analysis (Fig. 1c).

### Investigation of PLAC1 expression by prostate cancer cells

**Figure 2:**
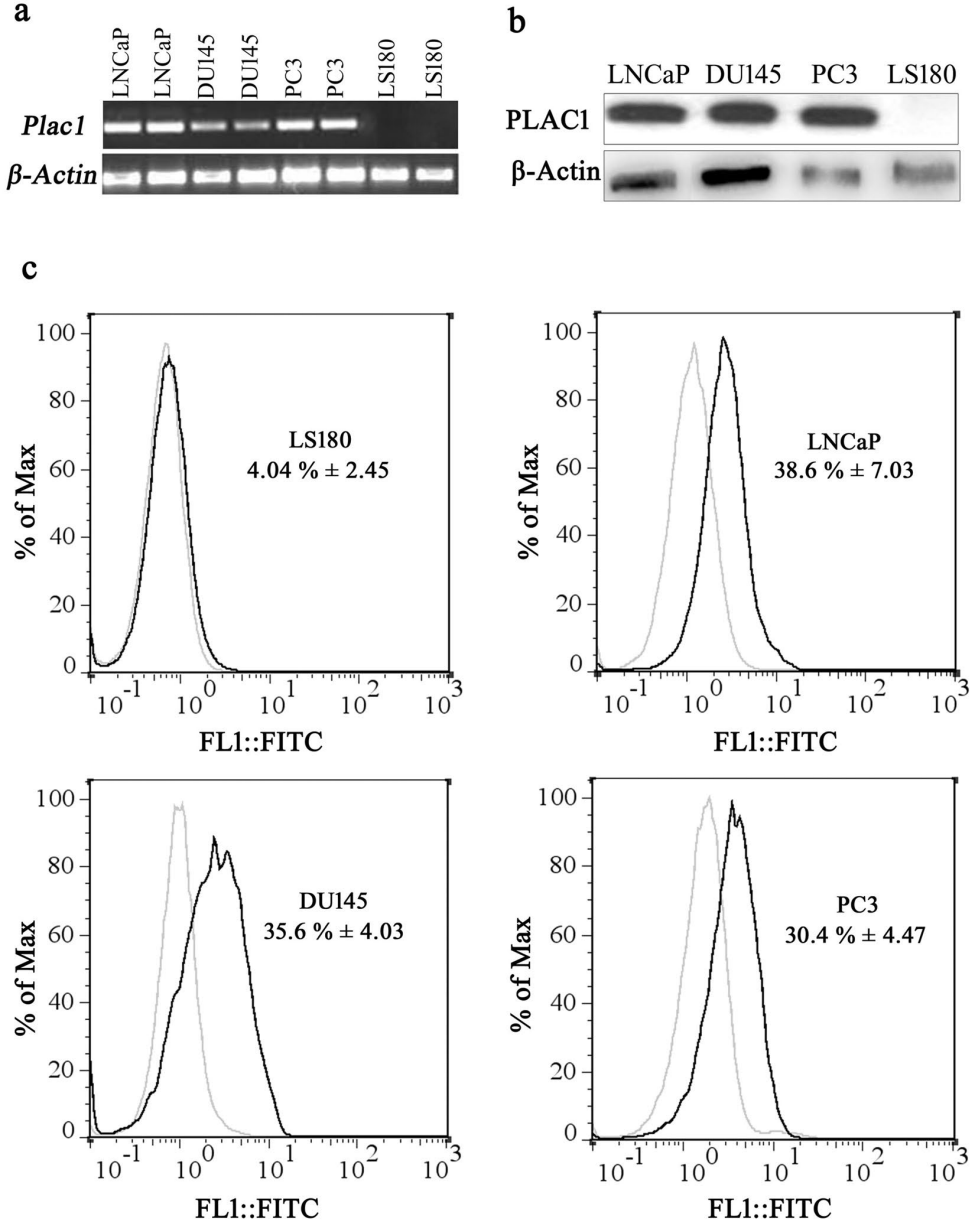
Characterization of PLAC1 expression by human prostate cancer cell lines: PLAC1 expression in prostate cancer cell lines LNCaP, DU145 and PC3 was assessed by RT-PCR (**a**) (full length gel in supplementary information file, Fig. S7a), Western blotting (**b**) (full length gel in supplementary information file, Fig. S7b) and flow cytometry (**c**). Human colon cancer cell line, LS180, was used as negative cell control. Optimal concentration of anti-PLAC1 antibody in flow cytometry experiments (2.5 µg/mL) was selected by applying a concentration range of 10 to 0.625 µg/mL antibody in LS180 cells. Beta actin was used as a loading control in RT-PCR and Western blotting. Data are representative of three experiments.

We first confirmed *Plac1* transcript expression in prostate cancer cell lines, LNCaP, DU145 and PC3 using RT-PCR. Colon cancer cell line, LS180, was used as a control and was always shown to be negative (Fig. 2a). In Western blotting, a distinct band of ~27 kDa was observed in all prostate cancer cell lines. As with RT-PCR, no expression of PLAC1 was observed in LS180 (Fig. 2b). Reactivity of the monoclonal anti-PLAC1 antibody, 2H12C12, with native target protein was tested in the next step by flow cytometry in prostate cancer cell lines. To determine optimal concentration of antibody leading to the specific signal, a concentration range of 10 to 0.625 µg/mL was probed in LS180 cells as negative control cells. In this case, a concentration of about 2.5 µg/mL was selected as the optimal concentration for further experiments in prostate cancer cell lines. In flow cytometry analysis of prostate cancer cells, 2H12C12 recognized surface PLAC1 in 38.6% ± 7.03, 35.6% ± 4.03 and 30.4% ± 4.47 of LNCaP, DU145 and PC3 cells, respectively (Fig. 2c).

### Preparation of anti-PLAC1-ADC

**Figure 3:**
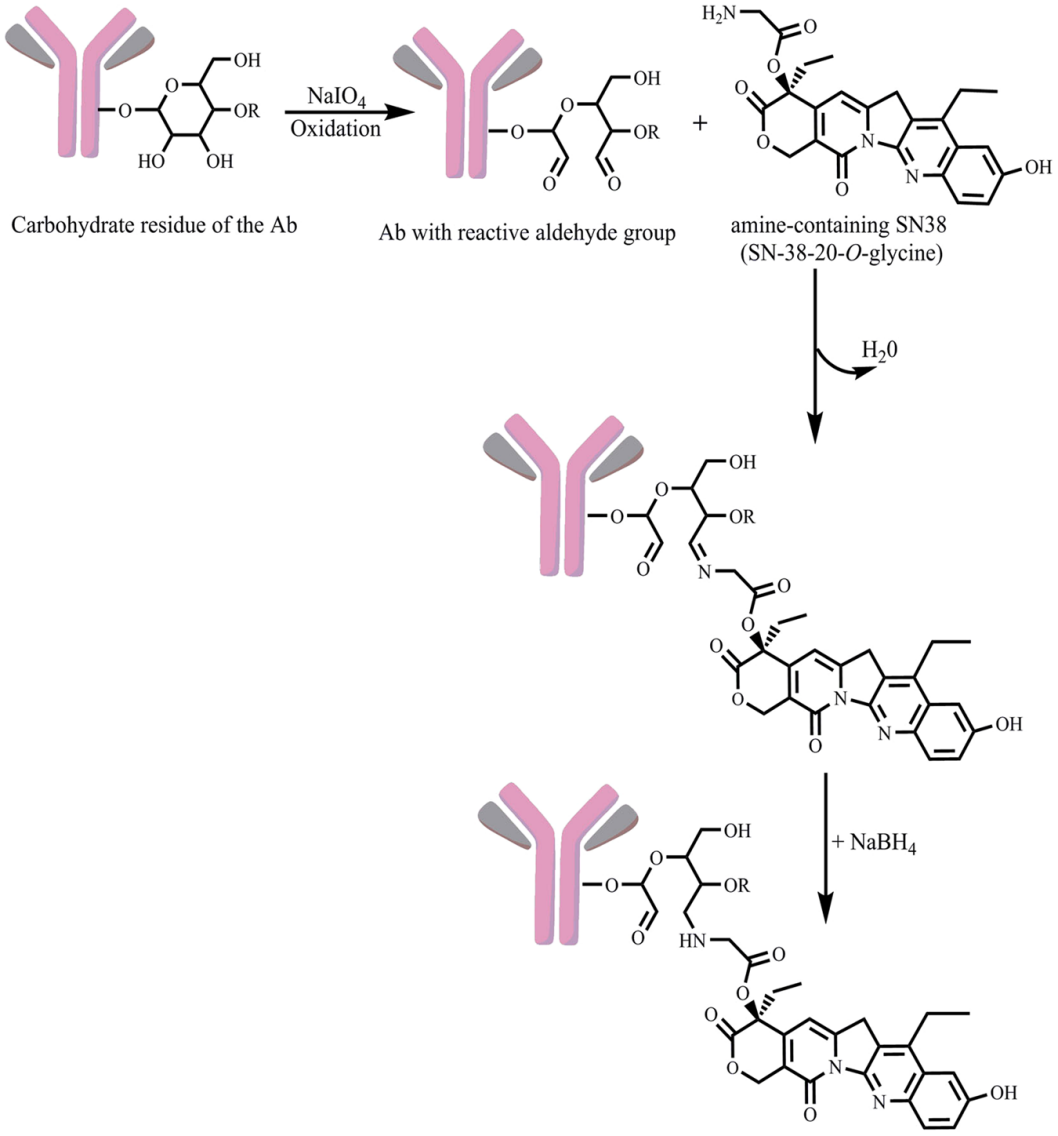
Scheme of SN38 conjugation to anti-PLAC1 antibody. SN38 was first modified as SN38–20-*O*-glycinate TFA salt to prepare linkable SN38 in three steps as descibed in materials and methods. Anti-PLAC1 antibody was oxidized with sodium metaperiodate and was reacted with SN38-20-*O*-glycine. The resultant conjugate was mixed with sodium borohydride to prepare stabilized secondary amine linkage (C-N).

Anti-PLAC1-ADC was generated by conjugation of 2H12C12 or Isotype-matched mouse immunoglobulin with aminated SN38. The structure of predicted products during synthesis of aminated SN38 was monitored and confirmed by Fourier transform infrared (FT-IR), ^1^H-NMR, and ^13^C-NMR spectra (Supplementary information file, Figs S1–S3). In our results, the FT-IR spectrum of the first step product shows the absence of an absorption band at the 3581 cm^−1^ region corresponding to the stretching vibrations of the phenolic -OH groups indicating the success of the protecting step in this site of the drug. Additionally, our results in ^1^H-NMR spectra showed a singlet signal at δ = 1.88 ppm with nine integral values indicating the presence of three −CH_3_ protons of BOC-group in protection step, and also a signal at δ = 3.95 ppm with two integral values indicating the presence of –CH_2_ protons of BOC-Glycine group (Figs S1 and S2), while the aforementioned signal was eliminated in deprotection step (Fig. S3). In addition, in the amine group formation step, the signal at δ = 1.88 ppm with two integral values indicates the presence of −NH_2_ protons of an amine group as determined by ^1^H-NMR spectroscopy (Fig. S3). Further, the increase and decrease in signal numbers as detected by ^13^C-NMR spectra represent an agreement with the carbon numbers in predicted structures of all steps that confirmed our expected structures. As shown in Fig. 3, oxidized carbohydrate residues of the antibodies provided reactive aldehyde groups for conjugation with amine-containing SN38 (SN38–20-*O*-glycine) through formation of Schiff base (imine) linkage. Subsequently, this relatively labile imine linkage was stabilized by reduction to a secondary amine linkage with sodium borohydride. Knowing that at alkaline pH, lactone E-ring of SN38 may be converted to an open carboxylate form impairing its cytotoxicity effect^19^, we carried out the schiff base cross-linking at a neutral pH (7–7.5). Setting the above pH for the conjugation process offered a controllable drug-antibody conjugation system. Drug-antibody ratio was measured by HPLC and was found to be 5.5 ± 0.5 representing the results of three independent experiments.

### Comparative determination of affinity constant of anti-PLAC1 antibody and its ADC

**Figure 4:**
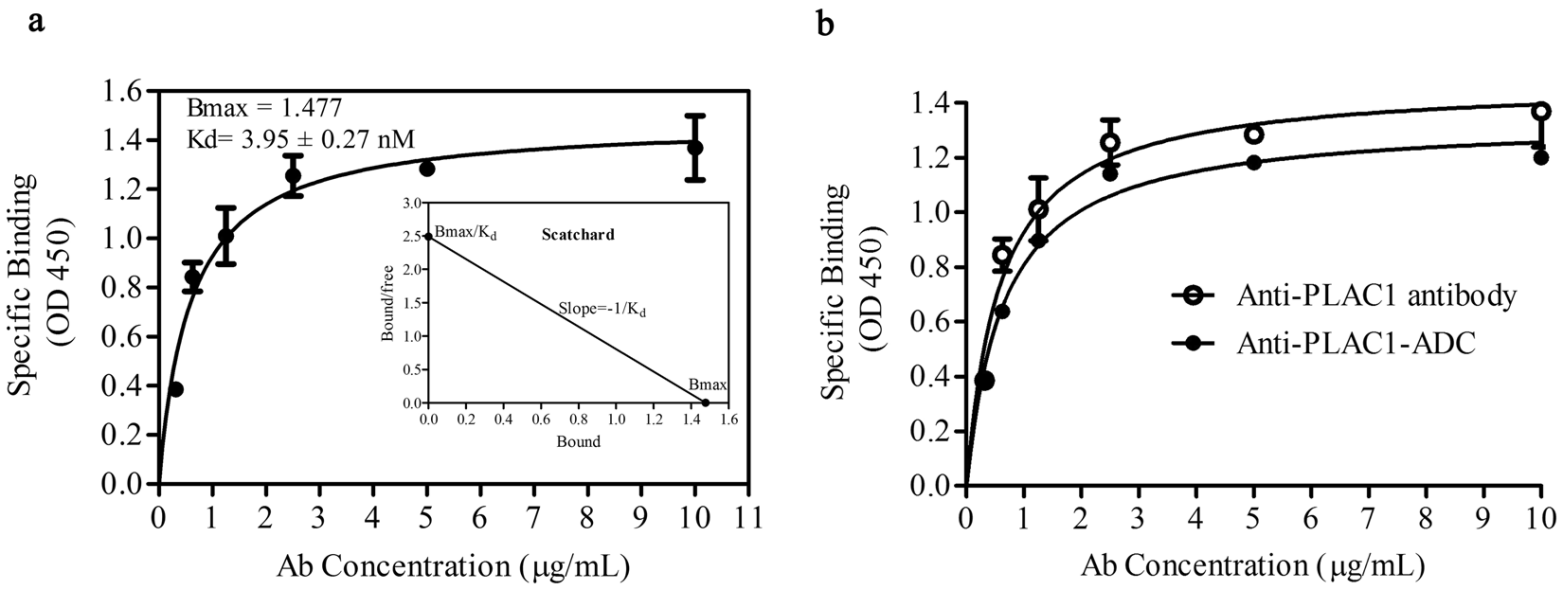
Saturation binding curve and scatchard plot of anti-PLAC1 antibody: Saturation binding curve was generated using the Prism™ software and the equation for one-site binding model [Y = Bmax*X/(Kd + X)], describing the equilibrium binding of the 2H12C12 antibody to the rhPLAC1 as a function of increasing antibody concentration (**a**). Comparative graphs depicting the Kd values (affinity constant) of anti-PLAC1 antibody and its ADC. Apparent Kd of anti-PLAC1 antibody and its ADC to the rhPLAC1 were compared and plotted with Graphpad Prism software by nonlinear regression using a one-site binding model (**b**). Data were generated from three independent experiments.

Binding data obtained from affinity constant experiment were entered into the Graphpad Prism software and analyzed based on nonlinear regression of a one-site specific binding model (Fig. 4). Based on this, the affinity constant of unconjugated 2H12C12 was shown to be 3.95 ± 0.27 nm (Fig. 4a). Scatchard plots of specific binding obtained with the Prism GraphPad program. The scatchard plot was shown to be linear indicating that 2H12C12 binds to a single epitope in PLAC1 (Fig. 4a).

To test whether conjugation of SN38 to the antibody affects its affinity to the cognate antigen, binding of anti-PLAC1-ADC and naked antibody to the coated PLAC1 was examined and compared. As shown in Fig. 4b, binding affinity of 2H12C12 was not significantly affected by SN38-conjugation process (3.95 ± 0.27 nM *vs*. 4.4 ± 0.27 nM, respectively).

### Comparative determination of internalization capacity of anti-PLAC1 antibody and its ADC

**Figure 5:**
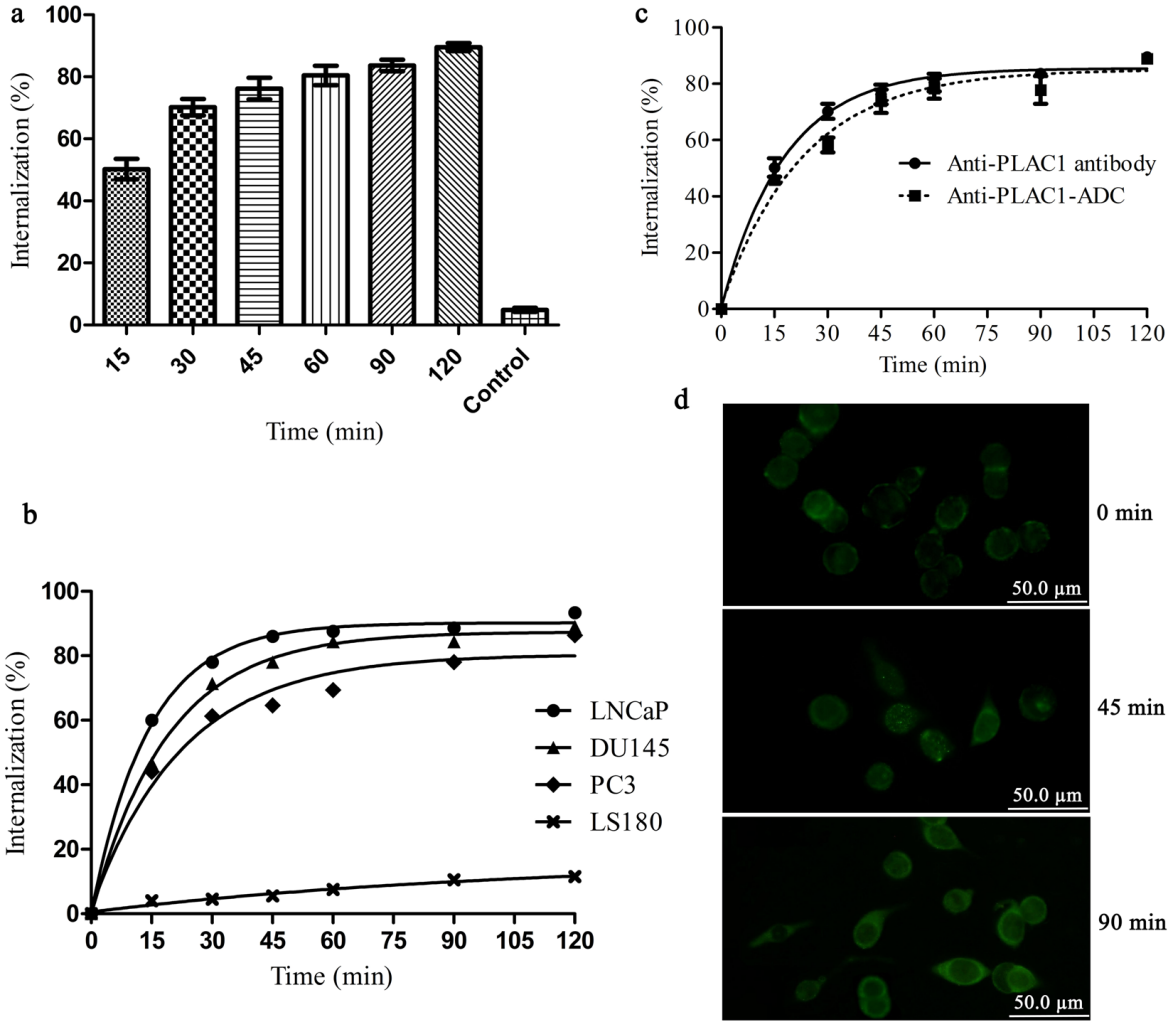
Comparative determination of internalization capacity of anti-PLAC1 antibody and its ADC. The degree of PLAC1 mAbs internalization in DU145, LNCaP, and PC3 cells was investigated by flow cytometric method at various time points. After each time point, the proportion of internalized fraction was measured (**a**). Comparative internalization of anti-PLAC1 antibody following binding to the surface PLAC1 is shown in LNCaP, DU145 and PC3 prostate cancer cells. LS180 was assessed in parallel as PLAC1 negative cell line (**b**). Internalization of anti-PLAC1 antibody and its SN38 conjugate after engagement with surface PLAC1 in LNCaP cells was tested and compared (**c**). Data were generated from three independent experiments. Internalization of anti-PLAC1 antibody in LNCaP cells was visualized at different time periods by immunofluorescent staining. A progressive accumulation of the fluorescent signal within cells indicates antibody internalization (**d**).

The degree of PLAC1 mAbs internalization in DU145, LNCaP, and PC3 cells was investigated by flow cytometric method at various time points (i.e., 15, 30, 45, 60, 90, and 120 minutes). After each time point, the proportion of antibody remaining in the cell surface was visualized with flourochrome-labeled secondary antibody. As demonstrated in Fig. 5a, internalization was done very soon within few minutes after incubation of the cells with anti-PLAC1 antibody and reached to about 50% after 15 min. The process of internalization was reached to the plateau level after about 45, 60 and 90 min in LNCaP, DU145 and PC3 cells, respectively. After 120 min, 93% ± 2.38, 89% ± 2.94 and 86% ± 3.62 of the antibody were internalized in cells mentioned above. To determine whether 2H12C12 internalization is mediated only by PLAC1, antibody was also incubated with LS180 cell as PLAC1 negative cell line. As shown in Fig. 5b, antibody was not internalized by this cell line indicating internalization is mostly dependent on receptor-mediated endocytosis pathway.

To further investigate if the process of SN38 conjugation to the anti-PLAC1 antibody affects its internalization property, internalization of anti-PLAC1 antibody and its SN38 conjugate after engagement with surface PLAC1 in LNCaP cells was tested in six separate experiments and compared. According to the readouts (Fig. 5c), no significant difference in internalization of anti-PLAC1 antibody and its ADC was observed. In order to visualize the routing of anti-PLAC1 antibody in prostate cancer cells, LNCaP cells were pulsed with the antibody for different times at 37 °C, then fixed and stained with FITC-conjugated secondary antibody. Thereafter, we visualized anti-PLAC1 antibody in LNCaP cell during the test period. The results showed a progressive internalization and accumulation of the fluorescent signal within cells indicating antibody-mediated PLAC1 internalization (Fig. 5d).

### Cytotoxicity profiling of anti-PLAC1-ADC in human primary prostate cancer cells and cell lines

**Figure 6:**
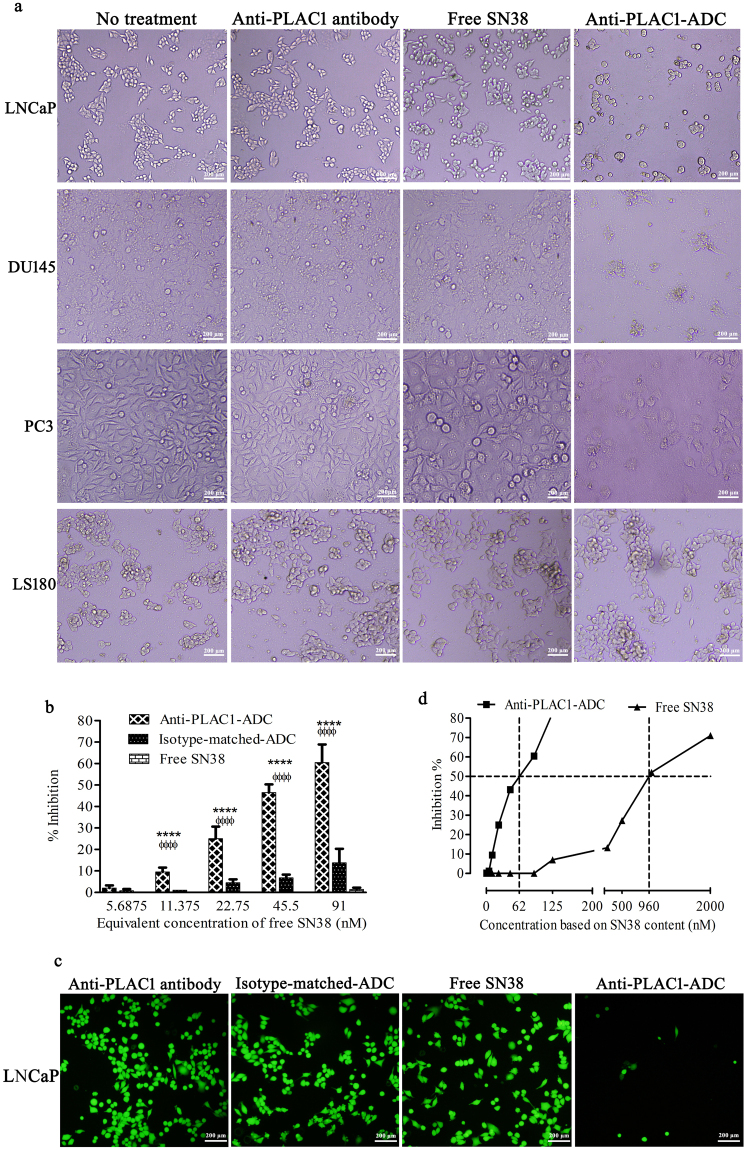
*In vitro* cytotoxicity assessment of anti-PLAC1-ADC. Prostate cancer cells and LS180, as negative cell control, were treated with 2.5 µg/mL anti-PLAC1 antibody or anti-PLAC1-ADC, equivalent concentration of free SN38, or remained untreated. Cell morphology was visualized after 48 h under microscope (**a**). LNCaP cells were treated with different concentrations of free SN38 or equivalent concentrations of anti-PLAC1-ADC or isotype-matched-ADC and the rate of cell cytotoxicity was assesses by Calcein AM fluorimetric assay (**b**). Calcein AM-labeled LNCaP cells were inspected under fluorescent microscope 36 h after treatment with 2.5 µg/mL anti-PLAC1-ADC, isotype-matched-ADC, anti-PLAC1 antibody or equivalent concentration of free SN38 (**c**). IC_50_ values for free SN38 and anti-PLAC1-ADC were determined using the Prism software as described in materials and methods (**d**). Data were generated from four independent experiments. *Anti-PLAC1-ADC *vs*. free SN38, ^ϕ^anti-PLAC1-ADC *vs*. isotype-matched-ADC, *or ^ϕ^p < 0.05, ** or ^ϕϕ^p < 0.01, *** or ^ϕϕϕ^p < 0.001, **** or ^ϕϕϕϕ^p < 0.0001.

**Figure 7:**
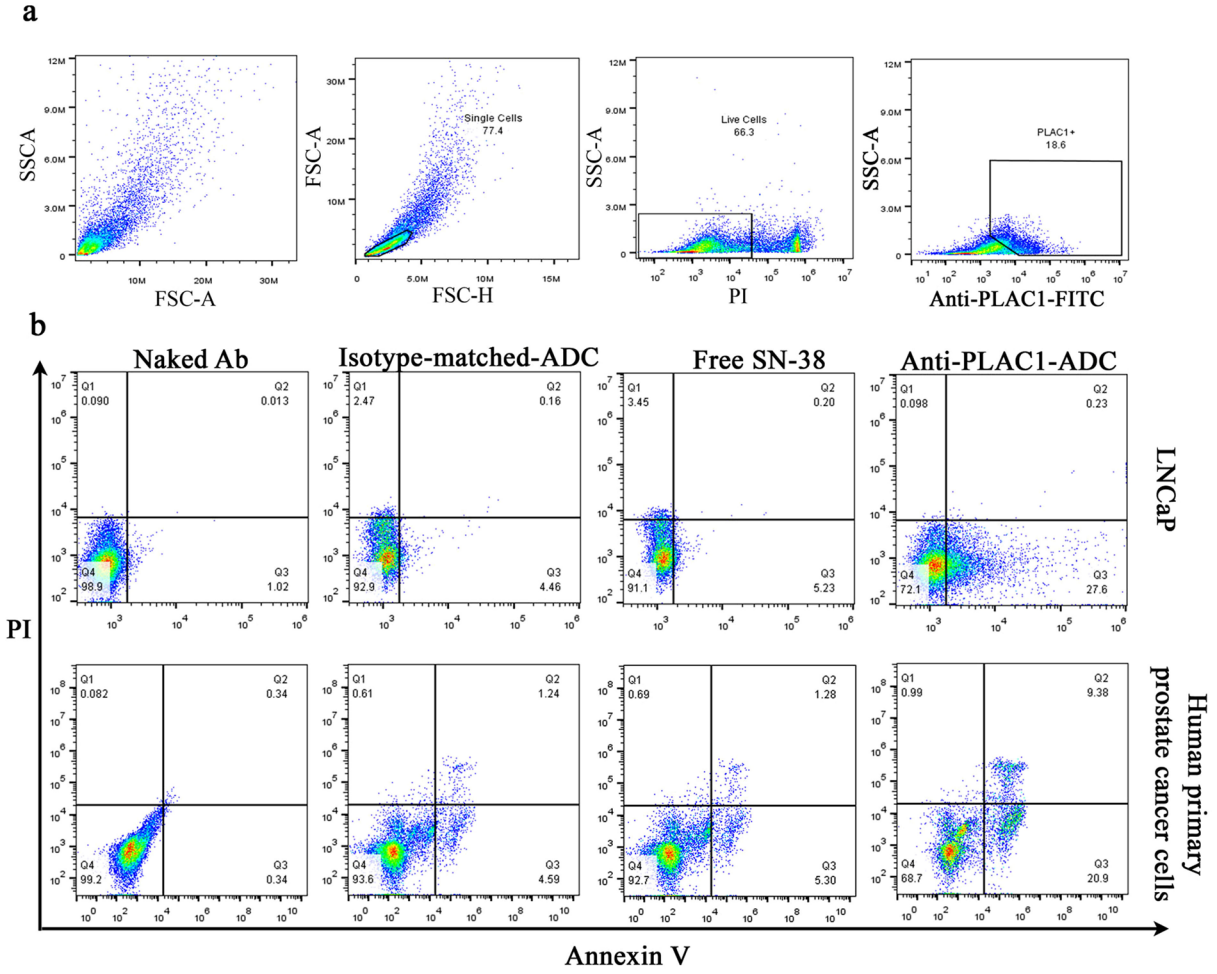
Effect of anti-PLAC1-ADC on induction of apoptosis in prostate cancer cells. Primary cultures of isolated epithelial cells of three human prostate cancer tissues were prepared and the level of PLAC1 expression was tested by flow cytometry (**a**). Pro-apoptotic activity of anti-PLAC1-ADC on LNCaP cells and human primary prostate cancer cells was assessed by Annexin V/Propidium Iodide (PI) apoptosis Assay (**b**). The results are representative of three experiments.

In a qualitative study, alteration in cell morphology following treatment was probed by inverted light microscopy. After a 48 h incubation period, anti-PLAC1-ADC efficiently induced cell death in a great proportion of prostate cancer cells (Fig. 6a), while no apparent cytotoxicity was observed in PLAC1 negative LS180 cell line. Indeed, PCa cells treated with equivalent concentration of either free SN38 or unconjugated antibody demonstrated no apparent changes in the cell morphology. The above observations were in agreement with quantitative study using cAM cytotoxicity assay in LNCaP cells. We found that conjugation of SN38 to the anti-PLAC1 antibody profoundly increased cytotoxic activity of SN38 against LNCaP cells compared to equivalent concentrations of free SN38 at all concentrations (p < 0.0001). In parallel, anti-PLAC1-ADC as compared with isotype-matched-ADC exerted significantly higher cytotoxic effects in LNCaP cells (p < 0.0001) (Fig. 6b). To confirm the aforesaid results, cAM-labled cells were inspected 36 h after treatment under fluorescent microscope to evaluate number of cells with positive green fluorescent signal. In line with the results mentioned above, we observed a clear reduction of fluorescing cells treated with anti-PLAC1-ADC compared to the cells treated with either isotype-matched-ADC, naked antibody or remained untreated (Fig. 6c). Anti-PLAC1-ADC was found to have an IC50 value of 1.7 µg/mL corresponding to about 62 nM SN38 which was 15-fold lower compared to that of free SN38 (960 nM) (Fig. 6d). In fact, free SN38 exerted no negligible cytotoxic effect till concentration 250 nM where only a small proportion of cells were killed, while, anti-PLAC1-conjugated SN38 showed substantial cytotoxicity starting from a concentration of about 23 nM (Fig. 6d). Cytotoxic effect of anti-PLAC1-ADC was further assessed by apoptosis assay in LNCaP and also in human primary prostate cancer cells. Three prostate cancer tissues with mean Gleason score of seven were tested. After isolation of epithelial cells, PLAC1 expression in three specimens was found to be 10%, 15% and 18.6% (Fig. 7a). The extent of apoptosis by anti-PLAC1-ADC corresponded to the percent of PLAC1 positive cells (Fig. 7b). In parallel, we found that about 30 ± 5% LNCaP cells undergo apoptosis after treatment with anti-PLAC1-ADC (Fig. 7b). Also, safety of anti-PLAC1-ADC was tested in Balb/c mice. The ADC-treated mice did not show weight loss (Fig. S4a) or other signs of toxicity during the period of follow up study. Indeed, in the pathological examination of eight tissues no apparent toxicity was observed (Fig. S4b).

## Discussion

Here for the first time we have reported the excellent potential of a novel antibody-drug conjugate for targeting PCa cells. Finding suitable targets specifically expressed by cancer and not normal cells is the main objective for cancer immunotherapy. Our findings clearly showed that in the majority of normal tissues including prostate no detectable levels of PLAC1 could be found^8,10^ pointing to the possibility of PCa targeted therapy by employing anti-PLAC1 antibodies. Based on Scatchard plot analysis, we showed here that anti-PLAC1 mAb, 2H12C12, reacted with its cognate ligand with high affinity (Kd~4 × 10^−9^ M) and a pattern indicative of a single population binding site. No reactivity was observed in control cells shown not to express PLAC1 indicating specificity of the antibody. Preliminary functional assessment revealed that 2H12C12, *per se*, does not exert much an effect on vital parameters of prostate cancer cells including cell cycle, adhesion and proliferation (data not shown). In contrast, Koslowski *et al*. showed that proliferation of breast cancer cell lines was markedly inhibited by rabbit anti-PLAC1 antibody^20^. This inconsistency can be explained by different cell lines investigated and also different polypeptides of PLAC1 molecule used in the two studies for generation of anti-PLAC1 antibody which results in interaction of the antibody with different epitopes.

Having in mind the PLAC1 specificity as well as rapid internalization profile of the antibody upon engagement with its cell surface ligand in prostate cancer cells prompted us to address therapeutic potential of a drug-conjugated antibody.

Target specificity and internalization are among the most important characteristics of a successful ADC^21–24^ in which the antibody functions as self-targeting nanocarrier^16,17^ enabling direct intracellular delivery of a cytotoxic drug to the tumor cells with minimal systemic toxicity and high therapeutic efficacy^22,23^. Based on the rationale behind the utility of ADCs, high tumor selective expression and low/null normal tissue expression pattern is the most critical requirement for an ADC^25^. Although most of the studies on PLAC1 expression have been oriented at the transcript level due to the uncertainty of available antibodies, the cumulative data indicate the differential overexpression of this marker in vast majority of solid tumors and cell lines^10,20,26–30^ making anti-PLAC1 antibodies a potentially suitable vehicle for delivering cytotoxic agents to cancer cells. In spite of the wide expression pattern of PLAC1 in variety of cancers, prostate cancer seems to be the prototype model for investigation of potential therapeutic effects of anti-PLAC1-based nanomedicine due to its high prevalence^1,31,32^ and differential expression of PLAC1 in PCa^8,33^.

Endocytic membrane activity of the target antigen and the capacity of its specific antibody to induce ligand internalization are among important variables in ADC research. In fact, the rate of internalization considerably affects the half-life of the ADC and the kinetics of the cytotoxic component^34^. We demonstrated using flow cytometry experiments that 2H12C12 has high antigenic specificity and undergoes rapid internalization upon binding to cell surface antigen. This finding was further confirmed by immunofluorescent staining demonstrating rapid accumulation of fluorescent signal within cells. Such a rapid internalization kinetic together with high specificity could potentially compensate for low surface expression of PLAC1 and implies that 2H12C12 could function as a vehicle to deliver a drug payload to the PLAC1 positive cancer cells. Although about 90% of antibody was internalized after 2 h in all cell lines examined, the rate of internalization was found to be faster in LNCaP compared to DU145 and PC3 prostate cancer cells. This finding is in agreement with higher expression of PLAC1 in LNCaP cells.

Selecting appropriate drugs suitable for empowering mAbs is of utmost importance in ADC design^35–37^. Supertoxic drugs with IC_50_ values in the nanomolar range^14,21^ have been successfully used to make ADCs. Well-characterized cytotoxic pathways and clearance mechanisms are more important features of drugs for ADC utility than merely their supertoxic nature. Among the supertoxic drugs, only SN38 has FDA approval for clinical use. It is a well-characterized CPT derivative acting through inhibition of topoisomerase I activity^38^ and possesses 100 to 1000-fold greater cytotoxicity compared to the parent compound^39^ making this derivative a very useful and potent anti-cancer drug. Indeed, SN38 has been successfully employed for targeting tumor initiating cells which could potentially reduce the likelihood of emergence of drug resistant tumor types^40^.

Conjugation chemistry has a significant impact on the therapeutic properties of ADCs. Although, current antibody-drug conjugation strategies are mainly based on the linkage of a cytotoxic drug to the antibody *via* primary amines (−NH_2_) or sulfhydryl (−SH) groups^37^, we aimed at to link SN38 to antibody *via* carbohydrate residues containing cis-diols(−CHO). This conjugation strategy provided an opportunity to directly link the cytotoxic agent to the antibody, avoiding the laborious linker-dependent conjugation step. Furthermore, direct linkage of cytotoxic agent to the antibody has been shown to result in more cytotoxic component stability in circulation than strategies employing a linker mediator^34^. On the other hand, linking drugs through carbohydrate residues which are mainly located in Fc region of the antibodies are less likely to impair binding capacity of antibody to its cognate epitope^41^. In line with this explanation, we found that neither affinity nor internalization capacity of anti-PLAC1 antibody in all cell lines tested was adversely affected by SN38 conjugation.

*In vitro* cytotoxic assay of anti-PLAC1-ADC against prostate cancer cell lines revealed consistent IC_50_ values in the lower nanomol range. In contrast, free SN38 exhibited an IC_50_ value about 15-fold higher than its corresponding ADC pointing to the fact that ADCs could profoundly improve therapeutic index of pharmaceutical drugs. Thus, it seems that enhanced ADC efficacy depends on its internalization and releasing SN38 inside the cells and not owing to free SN38 that are probably released outside the cell lines. Negligible cytotoxicity was observed when anti-PLAC1-ADC was incubated with LS180 cell line as PLAC-1 negative cells. This trend was also observed when isotype-matched-ADC control incubated with the PLAC1 positive cells, confirming that the ADCs cytotoxic potency is based on targeted drug delivery system. These results were also confirmed by apoptosis assay in LNCaP and human primary cancer cells. Our data showed that the extent of apoptosis by anti-PLAC1-ADC corresponded to the percent of PLAC1 positive cells pointing to the selective action of anti-PLAC1-ADC on cancer cells. In this regard, results of the experiments to assess safety and off-target effects of the anti-PLAC1-ADC showed no sign of morphological or histological toxicity in treated animals. Nonetheless, further experiments are needed to rule out toxicity of the prepared ADC.

In summary, here we showed that 2H12C12, a mAb directed against PLAC1, reacts with its cognate epitope with high affinity and enforce receptor internalization following engagement. Anti-PLAC1-ADC showed selective cytotoxicity against PLAC1-expressing prostate cancer cells and profoundly increased potency of cytotoxic drug payload suggesting PLAC1 as a highly promising therapeutic target for ADC-based cancer immunotherapy. To our knowledge, the ADC reported here is the first one to employ a PLAC1-specific antibody to deliver a cytotoxic agent, SN38, to tumor cells. We believe that the proposed anti-PLAC1-based ADCs may be a potent therapeutic modality against advanced PCa disease. Further evaluations are needed to determine its therapeutic potential and safety.

## Methods

### General Methods and Materials

All information regarding the solvents and methodologies for characterization of ADC is provided in supplementary information file.

### Cell lines, animals and tissues

Experiments on animals and human tissues were performed after approval of the research ethics committee of Avicenna Research Institute (ARI). All participants signed informed consent form before enrolling to this study. All methods were performed in accordance with the relevant guidelines and regulations. Five to seven weeks male Balb/c mice were prepared from animal facility of ARI. Tissues of prostate cancer were obtained from Hasheminejad Kidney Center (Tehran, Iran). The human prostatic cancer cell lines (LNCaP, DU145, and PC-3) were obtained from National Cell Bank of Iran (NCBI) (Pasteur Institute of Iran). Cell lines were cultured in Roswell Park Memorial Institute (RPMI 1640) medium (Gibco, USA) containing 10% FBS, 100 U/mL penicillin and 100 μg/mL streptomycin in a humidified atmosphere containing 5% CO_2_ at 37 °C. For each individual experiment, cells were not sub-cultured more than four passages.

### Production of recombinant PLAC1

The recombinant full length extracellular domain of human PLAC1 (rhPLAC1) was produced as described elsewhere^42^. More information is provided in supplementary information file.

### Generation and characterization of anti-PLAC1 antibodies

Anti-PLAC1 rabbit polyclonal was generated as described previously using rhPLAC1 as immunogen^10^. Based on the results obtained during screening and characterization steps, one clone designated as 2H12C12 was selected for subsequent ADC experiments. The monoclonal and polyclonal antibodies were purified from either mouse ascites fluid or rabbit sera by protein G affinity chromatography^24^ (Amersham Biosciences, the Netherlands). Antibody concentration was measured spectroscopically at 280 nm and the purity of the antibody was evaluated by SDS-PAGE. Reactivity of purified Abs was assessed by enzyme-linked immunosorbent assay (ELISA) using rhPLAC1 as coating layer. Reactivity of the antibodies was also tested by Western blotting as described elsewhere using lysate of human term placenta as positive control^10^ (Supplementary information file).

### Analysis of PLAC1 expression in prostate cancer cell lines

The expression of PLAC1 at gene and protein levels in prostate cancer cell lines was evaluated by PCR, Western blotting and flow cytometry. Colon cancer cell line, LS180 was included in all aforesaid experiments as negative cell control. For Western blotting, the same procedure as described above was employed except that the membranes were only probed with polyclonal rabbit anti-PLAC1 antibody. For PCR analysis, cDNA first strand reverse transcription was performed as published earlier^43^. PCR was carried out in a 25 μL volume with 12.5 μL master mix (Ampliqon, Denmark), 0.7 µL of each *Plac1* primer sets (10 µM) (Table 1, Fig. S5) and one microliter of cDNA. Reaction tubes were incubated in a thermocycler (Eppendorf, Germany) with the following thermal profile: 94 °C for 5 min for initial denaturation, 36 cycles of 94, 64, and 72 °C each for 30 seconds and final extension at 72 °C for 10 min. Primers were designated to amplify 364 bp fragment of *Plac1* mRNA. Amplification of β-Actin was used as internal control with ampliqon size of 174 bp. PCR products were electrophoresed on 1.5% agarose gel and the amplified bands were visualized and documented by UV transilluminator (UVP, USA). Surface expression of PLAC1 in prostate cancer cells was evaluated by flow cytometry using clone 2H12C12 as primary (2.5 µg/mL) and 1:100 dilution of FITC-conjugated sheep anti-mouse IgG (Sina biotech, Iran) as secondary antibody (Supplementary information file). Cells were then analyzed by flow cytometry.

**Table 1:** The sequence of forward (F) and reverse (R) primers used in this study.

Primers		Sequence	Product Length(bp)	Reference gene information
*Plac1*	F	5′-CAGTGAGCACAAAGCCACATTTC-3′	364 bp	NM_021796.3
	R	5′-CCACATTCAGTAACACGGTAGGTG-3′		
*rPlac1*	F	5′-AATTACATATGCAAAGTCCAATGACTGTGCTGTG-3′	570 bp	NM_021796.3
	R	5′-ATATAAGCTTTCACATGGACCCAATCATATCATC-3′		
*β-Actin*	F	5′-AGCCTCGCCTTTGCCGA–3′	174 bp	NM_001101.3
	R	5′-CTGGTGCCTGGGGCG–3′		

### Chemical modification of SN38 to achieve antibody-linkable functional group

7-Ethyl-10-hydroxycamptothecin (SN38) was modified as SN38–20-*O*-glycinate TFA salt to prepare linkable SN38^19^. This modification was carried out by a three step procedure of conversion to BOC-SN38, ester formation using BOC-glycine, and removal of BOC groups, as previously described elsewhere^44^ with minor modifications (Supplementary information file).

### ADC preparation and formulation

Drug-antibody conjugation protocol was a template of a method described by Wilson & Nakane 1978^45^ with the modification as follows. 0.5 mL of 1.0 mg/mL anti-PLAC1 antibody (clone 2H12C12) or isotype-matched negative control antibody were mixed separately with 10 µL of freshly prepared 0.1 M sodium metaperiodate and mixed for 20 min at RT protecting it from light. Excess oxidant was removed by dialyzing against 1 mM sodium acetate buffer (5 × 300 mL), pH 5.0 in the dark at 4 °C. The oxidized antibody was reacted with 1.5 µL (0.05 nmol) of the SN38-20-*O*-glycine (15 mg/mL) and immediately the pH was adjusted to 7.0–7.5 by adding 2 µL of 0.5 M fresh sodium bicarbonate buffer, pH 9.5. The solution was incubated on a roller mixer at 4 °C for 11 h. The resultant conjugate was mixed by 45 µL of freshly prepared sodium borohydride solution (4 mg/mL) and incubated at 4 °C on a roller mixer for 4 h to prepare stabilized secondary amine linkage (C-N). Upon completion of the conjugation reaction, excess drug was removed by dialyzing against PBS pH 7.0 at 4 °C and subsequently was confirmed by HPLC. The average drug-loading distribution (drug-antibody ratio, DAR,) of the prepared ADCs was determined by HPLC. Initially the pure SN38–20-*O*-glycine UV spectrum at 200–500 nm was obtained to determine drug λ max of 377 nm. Standard curve was plotted using different concentrations of the pure drug and determination of area under curve (AUC) of HPLC chromatograms at 377 nm. Then the concentration of SN38 in ADC samples was calculated using the calibration curve and its linear regression equation. The AUC of ADCs samples were measured at both 280 and 377 nm to make correction for probable contribution of drug absorbance at 280 nm. Based on the drug contents in ADC samples, DAR was calculated individually for each ADC preparations. The ADCs was then filter sterilized through a 0.22 µm syringe filter and stored at −20 °C.

### Determination of binding affinity (Kd) of anti-PLAC1 antibody

The affinity of anti-PLAC1 mAb to recombinant PLAC1 antigen was evaluated by a non-competitive ELISA^46^. Briefly, recombinant PLAC1 antigen at 10 or 5 µg/mL was plated into a 96 wells microplate and incubated overnight at 4 °C. The wells were washed 3 times with PBS-T for 3 min and then blocked with 3% skimmed milk in PBS-T at 37 °C for 90 minutes. After washing with PBS-T, anti-PLAC1 mAb was serially diluted 1:2 and added in triplicate, yielding a concentration range from 1 to 33.33 nM and incubated for 90 min at 37 °C. The wells were washed as above followed by addition of sheep-anti-mouse HRP conjugated secondary antibody (1:1500 dilution) for 90 minutes at 37 °C and signal development with TMB. Data were plotted as specific binding (OD_450_) versus anti-PLAC1 concentration and fitted to a one-site binding model and scatchard plot [Y = Bmax × X/(Kd + X)] using GraphPad Prism software. The Kd for ADC was determined in parallel using the same method.

### Antibody-mediated internalization studies

Flow cytometric assay was employed as described elsewhere^47^. In flow cytometric-based internalization assay, staining with primary antibody was done exactly as for determination of surface expression of PLAC1 in prostate cancer cells. After then, cells were incubated at 37 °C for different time points of 0, 15, 30, 45, 60, 90, and 120 minutes followed by 30 min treatment with secondary FITC-conjugated antibody at 4 °C to visualize Residual Mean Fluorescent Intensity (RFMI). The percentage of internalization was determined using the formula: 1 −(RMFI at 37 °C)/(RMFI at 4 °C)] $\times$ 100. RMFI at 4 °C represents MFI of the sample at the time point zero. The same approach was also employed for calculation of internalization of SN38-conjugated anti-PLAC1 antibody. Internalization of the anti-PLAC1 antibody was also tested by immunofluorescent staining (Supplementary information file).

### *In vitro* cytotoxicity study

Cytotoxic effect of anti-PLAC1-ADC was examined by qualitative and quantitative approaches. In qualitative cytotoxicity assay, cells were seeded in 96-well plates at the density of 1.0 × 10^4^ for LNCaP and 6.0 × 10^3^ for PC3 and DU145. Following overnight culture, the cells were treated in tetraplicate with serially diluted anti-PLAC1-ADC, isotype-matched-ADC, naked unconjugated anti-PLAC1 mAb (final concentrations of 0.156–2.5 μg/mL at final volume of 150 μL), or equivalent concentrations of free SN38. The cells were incubated at 37 °C in a humidified atmosphere containing 5% CO_2_ for 24, 42 and 72 h and cell integrity was inspected and photographed by an invert CKX41-Olympus microscope (Olympus Life Science, Japan). Quantitative cytotoxic assay was performed by a fluorimetric method using calcein-acetoxymethyl (cAM) (BD Biosciences Pharmingen, USA) dye. To this end, optimal dye concentration and cell seeding density was first determined in 96-well black wall plates (Corning, USA) to achieve optimal sensitivity of the test. Then the cells were treated as above for 36 h. Following washing with PBS, 100 µL of 5 µM cAM or PBS (as vehicle) was added to each well and the plates were incubated in CO_2_ incubator for 30 min. The extent of fluorescence intensity which is proportional to the number of viable cells was then measured by 1420 Multilabel fluorimeter Counter (PerkinElmer, USA) with excitation and emission wavelengths of 485 and 535 nm, respectively. Percent of cell cytotoxicity was calculated for each treatment by the following formula: %Inhibition = 100−[(Corrected mean fluorescent of sample)/(Corrected mean fluorescent of solvent Control) × 100], where corrected mean fluorescent was calculated as the average of fluorescent readout of each well subtracted from fluorescent readout of wells treated with Triton × 100. IC_50_ value were determined using the GraphPad Prism software and the equation for log (inhibitor) vs. response, Y = Bottom + (Top−Bottom)/(1 + 10^((LogIC_50_−X)*HillSlope)). In another setting, *in vitro* cytotoxicity of anti-PLAC1-ADC in human primary prostate cancer cells was tested as described below.

### Apoptosis assay

Human primary cultures were prepared from prostate cancer tissues (Supplementary information file). LNCaP and human primary prostate cancer cells were cultured at a density of 2 × 10^5^ cells/well in 24-well plates and treated with 2.5 µg/mL anti-PLAC1, anti-PLAC1-ADC or isotype-matched-ADC for 24 h at 37 °C. Control wells received the equivalent concentration of free SN-38. Cells were detached and stained with Annexin V-FITC and Propidium Iodide (PI) (Miltenyi Biotec, USA) according to the manufacturer’s instruction. Samples were analyzed by PartecPASIII flow cytometer (Partec GmbH, Germany).

### *In vivo* cytotoxicity study

Safety of anti-PLAC1-ADC was evaluated in *in vivo* animal experiments (Supplementary information file).

### Statistical analysis

All of the experiments were repeated at least three times with the same settings. Graphs were prepared using GraphPad Prism 5 software (Advanced Graphics Software, CA). Data were expressed as mean ± SEM. Statistical difference of Kd values, internalization capacity of unconjugated anti-PLAC1 antibody ant its SN38-conjugated ADC and weight of test and control groups in *in vivo* cytotoxicity experiments was determined by Mann Whitney test. Cytotoxic effects of anti-PLAC1-ADC, free SN38 and isotype-matched-ADC were analyzed with kruskal wallis and bonferroni multiple comparisons. The differences were considered to be significant at levels of p ≤ 0.05.

### Data availability

All data generated or analysed during this study are included in this published article (and its Supplementary Information file). More information is also available from the corresponding author on reasonable request.

## Electronic supplementary material

Supplementary Information
