# Supplementary material for: Placenta-specific1 (PLAC1) is a potential target for antibody-drug conjugate-based prostate cancer immunotherapy
Source: Sci Rep. 2017 Oct 17;7:13373. doi: 10.1038/s41598-017-13682-9 (PMC5645454; doi:10.1038/s41598-017-13682-9)
Supplement: Supplementary file 1 — Supplementary Information [file 41598_2017_13682_MOESM1_ESM.pdf]

Placenta-specific1 (PLAC1) is a potential target for antibody-drug conjugate-based prostate cancer immunotherapy

Mohammad-Reza Nejadmoghaddam[1&2], Amir-Hassan Zarnani[3,4], Ramin Ghahremanzadeh[2], Roya Ghods[5,6], Jafar Mahmoudian[1], Maryam Yousefi[2], Mahbobeh Nazari[7], Mohammad Hossein Ghahremani[8], Maryam Abolhasani[9], Ali Anissian[10,] Morteza Mahmoudi[1], Rassoul Dinarvand [1&11*]*

[1]*Nanotechnology Research Center, Faculty of Pharmacy, Tehran University of Medical Sciences (TUMS), Tehran, Iran.*

[2]*Nanobiotechnology Research Center, Avicenna Research Institute, ACECR, Tehran, Iran.*

[3]*Reproductive Immunology Research Center, Avicenna Research Institute, ACECR, Tehran, Iran.*

[4]*Immunology Research Center, Iran University of Medical Sciences, IUMS, Tehran, Iran.*

[5]*Oncopathology Research Center, Iran University of Medical Sciences, IUMS, Tehran, Iran.*

[6]*Department of Molecular Medicine, Faculty of Advanced Technologies in Medicine, Iran University of Medicine Sciences, IUMS, Tehran, Iran.*

[7]*Monoclonal Antibody Research Center, Avicenna Research Institute, ACECR, Tehran, Iran*

[8]*Department of Pharmacology-Toxicology, Faculty of Pharmacy, Tehran University of Medical Sciences(TUMS), Tehran, Iran.*

[9]*Department of Pathology, Hasheminejad Kidney Center, Iran University of Medical Sciences, IUMS, Tehran, Iran*

[10]*Veterinary department, Islamic Azad University, Abhar branch, Abhar, Iran.*

[11]*Department of Pharmaceutics, Faculty of Pharmacy, Tehran University of Medical Sciences, Tehran, Iran.*

***Correspondence:**

Amir-Hassan Zarnani, D.M.T., Ph.D.,

Reproductive Immunology Research Center, Avicenna Research Institute, ACECR, Tehran, Iran

Tel: +98 2122432020

Fax: +98 2122432021

E-mail: zarnania@gmail.com, zarnani@ari.ir

P.O. Box: 1936773493

Rassoul Dinarvand, Pharm D., PhD.,

Nanotechnology Research Center, Faculty of Pharmacy, Tehran University of Medical Sciences, Tehran, Iran.

Tel: +98 2164121014.

Fax: +982166959052.

Email: dinarvand@tums.ac.ir

P.O. Box: 1417614411

General Methods and Materials

All commercially available solvents were prepared from Sigma and Merck companies (USA), except 7-ethyl-10-hydroxycamptothecin (SN38) which was purchased from Gyro, Shanghai Puyi Chemical Co., Ltd (China). The purity determination of the substrates and reaction monitoring were accomplished by TLC on silica-gel polygram SILG/UV 254 plates (Merck). ^{1}H NMR and ^{13}C NMR spectra were recorded in DMSO–d_6 solvent on a Bruker DRX-400 spectrometer., chemical shifts (δ) are expressed in parts per million (ppm) and are reported as s (singlet), d (doublet), t (triplet) and m (multiplet); coupling constants, J, are given in Hertz (Hz). IR spectra were recorded as KBr pellets on a Perkin-Elmer 781 spectrophotometer and an Impact 400 Nicolet FT-IR spectrophotometer. Mass spectrometry was done on an Agilent Technologies 5975C VL MSD instrument with Tripe-Axis Detector (Agilent Technologies, USA). Analytical HPLC was performed on Smartline Knauer systems (Knauer, Germany) with a Grace C18 column (5 µm, 300 Å, 4.6×250 mm), and with gradient elution including buffers A(0.3 % w/v ammonium acetate, pH 4.43, filtered through a 0.45 µm) and B(9:1 v/v acetonitrile: buffer A) advised earlier by Moon et al., 2008[1] with some modifications as follows: 100% A changing to 100% B over 20 min (linear) at a flow rate of 0.7 ml/min. Flow cytometery assays were done using PartecPASIII flow cytometer (Partec GmbH, Germany), and to permit a reliable flow cytometer data, an average 10000-30000 cells were counted and analyzed by FlowJo 7.6.1 software (TreeStar Inc., OR).

Chemical modification of SN38 to achieve antibody-linkable functional group

7-Ethyl-10-hydroxycamptothecin (SN38) was modified as SN38–20-*O*-glycinate TFA salt to prepare linkable SN38[1]. This modification was carried out by a three step procedure of conversion to BOC-SN38, ester formation using BOC-glycine, and removal of BOC groups, as previously described elsewhere[8] with minor modifications. Briefly, to protect the phenolic

OH group, SN38, (1 gr, 2.55 mmol) was dissolved in anhydrous dichloromethane (DCM, 80 mL) and reacted under stirring, with di-tert-butyl dicarbonate (1.45 ml, 6.32 mmol) in the presence of anhydrous pyridine (12.4 mL) at room temperature (RT) to generate BOC-SN38. After 24 h, the solution was washed two times with 150 ml HCl (0.5 N), and two times with 150 mL saturated $NaHCO_3$, and then extracted with DCM. The organic phase was separated and then anhydrous sodium sulfate was added for drying organic phase then filtered and finally the solvent was evaporated under ambient condition to obtain pure BOC-SN38 (95% yield, 1.19 gr). ^1H NMR (DMSO-d_6, 300 MHz): δ ppm: 0.86-0.90 (3H, t, CH_3), 1.26-1.31 (3H, t, CH_3), 1.54 (9H, s, 3CH_3), 1.83-1.91 (2H, m CH_2), 3.18-3.20 (2H, m CH_2), 5.32 (2H, s, NCH_2), 5.44 (2H, s, OCH_2), 6.56 (1H, s, C=CH), 7.71-8.21 (3 H, m, ArH). ^{13}C NMR (DMSO-d_6, 75 MHz): δ ppm: 8.2, 14.3, 22.7, 27.7, 30.7, 49.9, 65.7, 72.8, 84.2, 97.1, 115.3, 119.5, 125.7, 127.4, 128.9, 131.7, 145.9, 146.2, 146.9, 149.7, 150.4, 151.5, 152.4, 157.2, 172.9 (Fig.S1).

Afterward, BOC-SN38 (600 mg, 1.22 mmol) was reacted with BOC-glycine (450 mg, 2.57 mmol), under stirring, in the presence of anhydrous DCM (15 mL), 1-Ethyl-3-[3-dimethylaminopropyl]carbodiimide hydrochloride (EDAC) (460 mg, 2.4 mmol), and 4-dimethylaminopyridine (DMAP) (159 mg, 1.24 mmol) for two weeks at ambient condition. The crude product was then transferred into a separatory funnel, washed and extracted with 25 ml of Milli-Q water (once), 25 mL 0.5 N HCl (twice), 25 ml saturated bicarbonate (twice), and 25 ml normal saline (once). Then anhydrous sodium sulfate was used for drying liquid organic phase, subsequently filtered, and finally the product was solvent evaporated to obtain powder. Then the powdery product was purified in excess amount of boiling methanol to gain pale yellow pure powder. Product structure at first was characterized based on ^1H NMR, and ^{13}C NMR spectra. In the ^1H NMR spectrum of product, the only one signal at δ= 1.37 ppm with nine integral values indicated the presence of three –CH3 protons of one BOC group.

The ^{13}C NMR spectrum showed 27 distinct signals. Surprisingly, in the purification step and in the presence of excess amount of hot methanol, the BOC group on C-10 was hydrolyzed and the process did not proceed according to expectation and spectral data were inconsistent with the expected structure BOC-SN38-glycine-BOC. Indeed, the data were in good agreement with the structure of an unprecedented product and showed the structure of the unexpected product SN38-glycine-BOC.

SN38-glycine-BOC, (91% yield, 0.61 gr). ^{1}H NMR (DMSO-d_6, 300 MHz): δ ppm: 0.93 (3H, t, CH3), 1.03 (3H, t, CH3), 1.37 (9H, s, 3CH3), 2.10-2.12 (2H, m CH2), 3.06-3.08 (2H, m CH2), 3.76-3.97 (2H, m, CH2NHBOC), 5.28 (2H, s, NCH2), 5.48 (2H, s, OCH2), 7.11 (1H, s, C=CH), 7.38-8.01 (3 H, m, ArH), 10.37 (1H, s, OH) .^{13}C NMR (DMSO-d6, 75 MHz): δ ppm: 8.0, 13.8, 22.7, 28.6, 30.7, 42.5, 49.9, 66.7, 76.7, 78.9, 95.1, 105.2, 118.2, 122.9, 128.4, 128.6, 131.9, 143.2, 144.1, 145.7, 147.3, 149.1, 156.3, 157.1, 157.2, 167.7, 170.0 (Fig. S2).

Deprotection of SN38-glycine-BOC was carried out by adding 200 mg (0.36 mmol) of this product to a mixture containing of 1.5 ml trifluoroacetic acid (TFA), 1.5 mL DCM, 0.083 ml anisole, and 0.042 mL water. The mixture was stirred at 30 °C for 90 minutes. Afterward, diethyl ether was slowly added to precipitate the product. The crude product was filtered and washed twice (2 × 50 mL) with diethyl ether. The resulting solid were dissolved in 20 ml of DCM, precipitated with 40 mL of diethyl ether, filtered, and washed with diethyl ether (2 × 20 mL) to obtain pure product. The product was dried under ambient condition to give SN38–20-*O*-glycine (yellow powder; 98% yield, 160 mg,). ^{1}H NMR (DMSO-d_6, 300 MHz): δ ppm: 0.92-0.97 (3H, t, CH3), 1.27-1.31 (3H, t, CH3), 2.16-2.22 (2H, m CH2), 3.08-3.13 (2H, m CH2), 4.07-4.37 (2H, m, COCH2), 5.32 (2H, s, NCH2), 5.54 (2H, s, OCH2), 7.18 (1H, s, C=CH), 7.41-8.03 (3 H, m, ArH), 8.42 (2H, brs, NH2), 10.50 (1H, brs, OH) (Fig.S3).

Production of recombinant PLAC1

The recombinant full length extracellular domain of human PLAC1 was produced as described elsewhere[2]. In brief, cDNA of *Plac1* (GenBank Accession No. NM_021796.3) was amplified from human placenta with the primers (*rPlac1*) listed in Table 1. Forward and reverse primers were designed to have Ndel and HindIII digestion sites at their 5' ends. The amplicon was digested with NdeI/ HindIII restriction enzymes and ligated in digested/dephosphorylated pColdI plasmid. After confirmation of cloning with DNA sequencing, the construct was transformed to Rosetta2 (DE3) competent cells. Full length recombinant protein was obtained in 1mM IPTG at 15 °C for 24h. rPLAC1 was expressed as inclusion bodies and solubilization was done with buffer containing Urea 2M, (50 mM Tris-HCl, 5 mM EDTA, 1 mM PMSF, and 2 M urea, pH 12). Protein purification with His6-tag in N-terminal was done with Ni sepharose™ high performance (GE healthcare, UK), according to the manufacturer's procedure. To investigate the identity of the recombinant protein, Western blot analysis was performed. Shortly, recombinant protein was electrophoresed and blotted to polyvinylidene fluoride (PVDF) membranes and probed with 1:100000 dilution of horseradish peroxidase (HRP)-conjugated monoclonal anti-His antibody (Roche applied science, Germany). Signal was developed by an ECL detection kit (GH Healthcare) according to the manufacturer's instruction.

Generation and characterization of anti-PLAC1 antibodies

Anti-PLAC1 rabbit polyclonal was generated as described previously using recombinant extracellular domain of human PLAC1 as immunogen[3]. Mouse monoclonal antibodies were generated in Balb/c mice by standard hybridoma technology using above-mentioned immunogen[4]. Based on the results obtained during screening and characterization steps, one clone designated as 2H12C12 was selected for subsequent ADC experiments. The

monoclonal and polyclonal antibodies were purified from either mouse ascites fluid or rabbit sera by protein G affinity chromatography (Amersham Biosciences, the Netherlands). Antibody concentration was measured spectroscopically at 280 nm and the purity of the antibody was evaluated by SDS-PAGE. Reactivity of purified Abs was assessed by enzyme-linked immunosorbent assay (ELISA) using recombinant human PLAC1 as coating layer. In brief, serial dilutions of the recombinant human PLAC1 from 0.15 to 10 µg/mL were coated in 96-well ELISA plate (Nunc, Denmark) and incubated overnight at 4 °C. The wells were washed 3 times with phosphate-buffered saline (PBS) containing 0.05% Tween 20, (PBS-T) for 3 min and then blocked with 3% skimmed milk in PBS-T at 37 °C for 90 minutes. Anti-PLAC1 antibody (2H12C12) at 5 µg/mL was added to the wells and subsequently incubated for 90 minutes. After washing, horseradish peroxidase (HRP)-conjugated sheep anti-mouse IgG (1:1500) (Sina biotech, Iran) was added to the wells and incubation was continued for furthered 90 minutes at 37 °C. Then, tetramethylbenzidine (TMB) (Sigma-Aldrich Chemie GmbH, Germany) was added to each well and the plates were incubated at room temperature in the dark. After 15 min, the reaction was stopped by adding 20% H_2SO_4 to the wells and optical densities (OD) were measured at 450 nm. Reactivity of the antibodies was also tested by Western blotting as described elsewhere using lysate of human term placenta as positive control[4]. To this end, lysates of fetal side of human term placenta obtained during cesarean delivery were prepared in a lysis buffer containing 20 mM Tris-HCl pH 8.0, 137 mM NaCl, 2 mM EDTA, 1% Triron X-100, 10% glycerol followed by determination of protein concentration using the bicinchoninic acid (BCA) method (Pierce BCA Protein Assay kit, Thermo Scientific, USA). Equal amounts of proteins from of cell lysates (20 µg) were resolved on 15% SDS polyacrylamide gels under non-reducing condition, electro-transferred to PVDF[5] and the membranes were treated with 50 ng/mL monoclonal or polyclonal rabbit anti-human PLAC1 antibody for 1.5 h. After washing, membranes were incubated with either

sheep anti-mouse or anti-rabbit IgG-HRP (Sina Biotech) for 1 h followed by signal development by ECL detection kit (GH Healthcare) according to the manufacturer's instruction. Beta actin was used as internal loading control using rabbit anti-human beta actin (Abcam, UK) and HRP-conjugated goat anti-rabbit IgG (Abcam) as primary and secondary antibodies, respectively.

Analysis of PLAC1 expression in prostate cancer cell lines

The expression of PLAC1 at gene and protein levels in prostate cancer cell lines was evaluated by PCR, Western blotting and flow cytometry. Colon cancer cell line, LS180 was included in all aforesaid experiments as negative cell control. For Western blotting, the same procedure as described above was employed using polyclonal rabbit anti-PLAC1 antibody as the primary antibody. For PCR analysis, cDNA first strand reverse transcription was performed as published earlier[6]. PCR was carried out in a 25 Ml volume with 12.5 µL master mix (Ampliqon, Denmark), 0.7 µL of each *Plac1* primer sets (10 µM) (Table 1) and one microliter of cDNA. Reaction tubes were incubated in a thermocycler (Eppendorf, Germany) with the following thermal profile: 94°C for 5 min for initial denaturation, 36 cycles of 94, 64, and 72 °C each for 30 seconds and final extension at 72 °C for 10 min. Primers were designated to amplify 364 bp fragment of *Plac1* mRNA. Amplification of β-actin was used as internal control with ampliqon size of 174bp. PCR products were electrophoresed on 1.5% agarose gel and the amplified bands were visualized and documented by UV transilluminator (UVP, USA). Surface expression of PLAC1 in prostate cancer cells was evaluated by flow cytometry using clone 2H12C12 as primary (2.5 µg/mL) and 1:100 dilution of FITC-conjugated sheep anti-mouse IgG (Sina biotech) as secondary antibody. Briefly, human prostatic cancer cell lines were grown to approximately 80% confluency and detached using pre-warmed citric-saline solution (citric acid 15mM, KCl 135 mM, pH 8.0), harvested, and

counted using the trypan blue exclusion method. The cells were washed twice in PBS containing 2% FBS (PBS-FBS). Monoclonal anti-human PLAC1, clone 2H12C12, was added to the cells at final concentration of 2.5 µg/mL for 45 min at 4 °C. Isotype-matched mouse immunoglobulin was used as isotype control. The cells were then washed as above followed by incubation with 1:100 dilution of FITC-conjugated sheep anti-mouse IgG (Sina biotech) for 30 min at 4 °C. Cells were then washed and analyzed by flow cytometry.

Immunofluorescence monitoring of antibody internalization

In brief, LNCaP cells were detached using citric-saline solution, washed and cultured in 6-chamber slides in a CO_2 incubator. After 24 h, cells were washed with warm PBS and then treated with anti-PLAC1 antibody (2.5µg/mL) diluted in cold PBS-FBS. After being incubated for 45 min at 4 °C, cells were washed with PBS-FBS three times. Reference cells (no internalization) were then immediately fixed with ice-cold acetone for 5 min and washed, while the other wells remained un-fixed. Slides were transferred to a 37 °C incubator for 45 and 90 min, after then un-fixed cells were fixed as above and washed. FITC-conjugated secondary antibody (Sina Biotech) at a 1:100 dilution was then applied to all wells for 45 min, after which wells were washed, mounted in PBS-glycerol and inspected under an immunofluorescent microscope (Olympus, BX51, Japan).

Tissue digestion and preparation of single cells

Methodology for prostate tissue digestion and preparation of single cells was adapted from the protocol published elsewhere[7] with some modifications. Radical prostatectomy specimens from three patients with approved prostate cancer were immediately transported after surgery to the laboratory in a cold transport buffer (RPMI 1640 with 10% FCS, 1% (v/v) penicillin-streptomycin, 0.5 µg/mL amphotericine B and 100 µg/mL gentamicin. A piece of tissue was

fixed in 10% (v/v) formalin for pathological examination. Tissues were washed in PBS containing 100 µg/mL gentamicin and were cut into pieces of about 2 mm^3. About 0.5 gram of diced tissues were placed into a sterile 50-mL tube and re-suspended in 3.5 mL transport medium containing 150 U/mL collagenase I (Gibco. USA). Digestion was continued for 10 h at 37 °C in an incubator with a rotor. After digestion, large clumps were broken by gentle pipetting. Cell suspension was then centrifuged at 800 g for 5 min at 4 °C. Pellet was washed two times with cold PBS at 800 g for 5 min at 4 °C and supernatant was discarded. Cells were then re-suspended in cold RPMI 1640 and centrifuged at 100 g for 1 min at 4 °C. Supernatant containing stromal cells was discarded and sediment containing acini was digested with 5 mL 0.1% (w/v) trypsin-EDTA for 20 min at a 37 °C water bath to obtain single epithelial cells. Trypsin activity was neutralized by FCS and cells were washed two times as above. Cell suspension was passed through a 40-µm cell strainer. Viability of the cells was tested by trypan blue exclusion. Cells were then cultured in KSF medium (Life technologies, USA) containing 10 ng/mL epidermal growth factor (Royan Institute, Iran), 2 mM L-glutamin and 1% (v/v) penicillin-streptomycin for 24 h. After then, expression of PLAC1 in isolated cells was examined by flow cytometry. In parallel, *in vitro* cytotoxicity of anti-PLAC1-ADC was tested as described in materials and methods and percent of apoptosis was measured.

In vivo cytotoxicity study

Eight male Balb/c mice at 5-7 weeks of age were randomly assigned to two groups and injected intraperitoneally twice at a one-week interval with 20 µg anti-PLAC1-ADC or the same dose unconjugated anti-PLAC1 for experimental and control groups, respectively. Mice were then closely monitored for symptoms of drug intolerability such as hair and weight loss, hunched posture, ruffled fur, diarrhea, and reduced movement. On day 30, the animals

were anesthetized with IP injection of a mixture of ketamine hydrochloride and xylazine hydrochloride and then dissected. Liver, lung, kidney, testis, stomach, bladder, spleen and skin of the animals were isolated, fixed in 10% natural buffered formalin and processed for histopathologic evaluation. Slides were then blindly reviewed by a pathologist to assess possible pathological effects of the drug.

Supplementary Figures and legends:

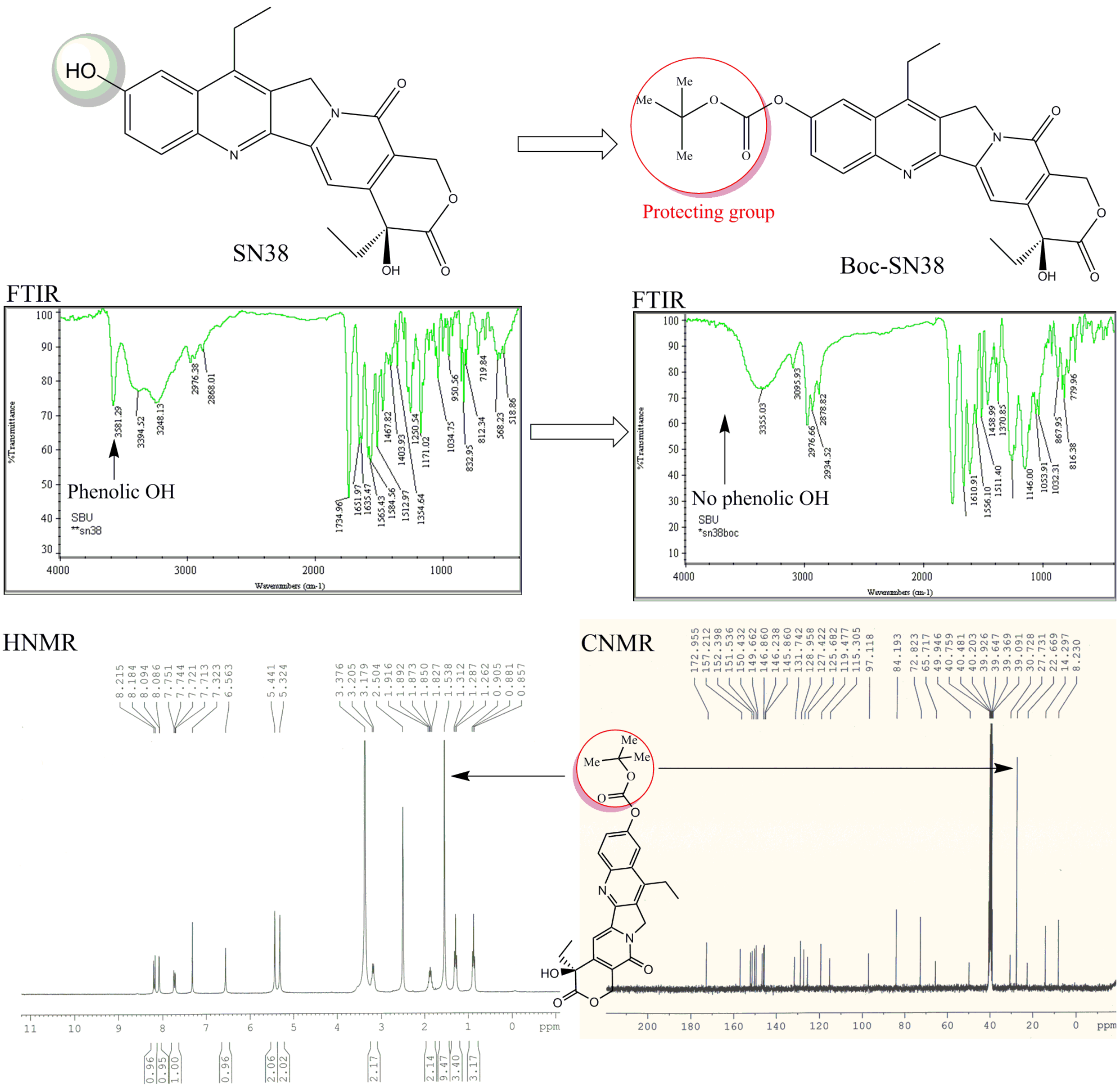

Fig. S1: Protection of SN38's phenolic OH group (Conversion of SN38 to BOC-SN38).

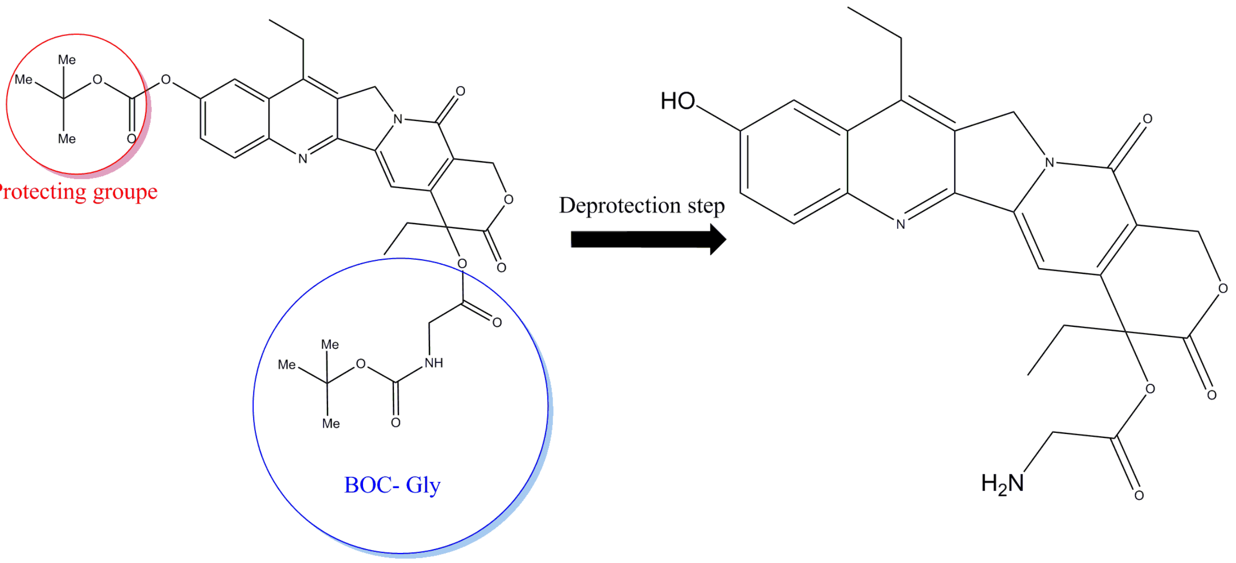

HNMR

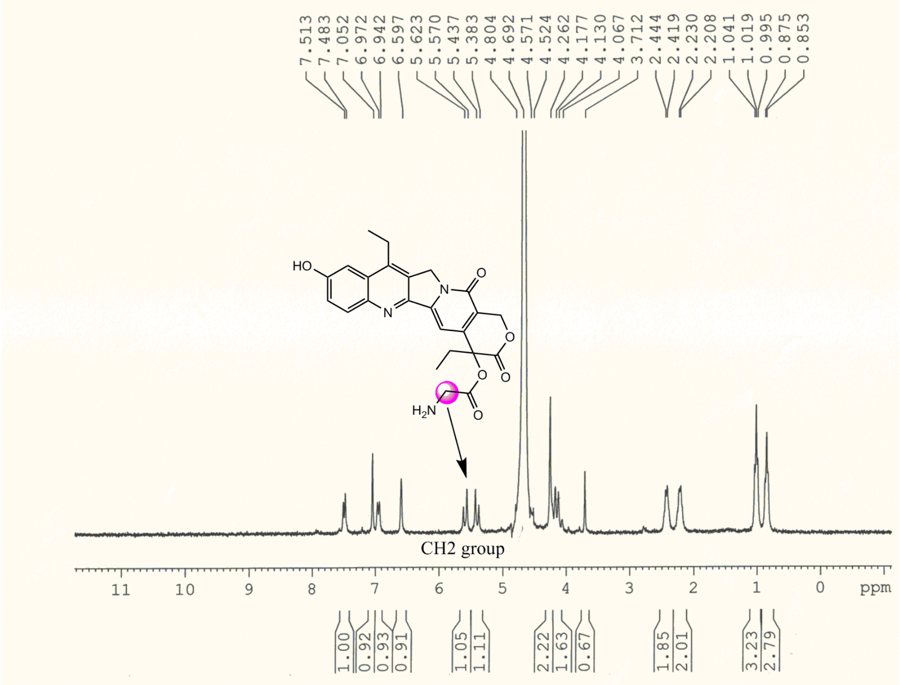

Fig. S2: Production of BOC-SN38-glycine-BOC.

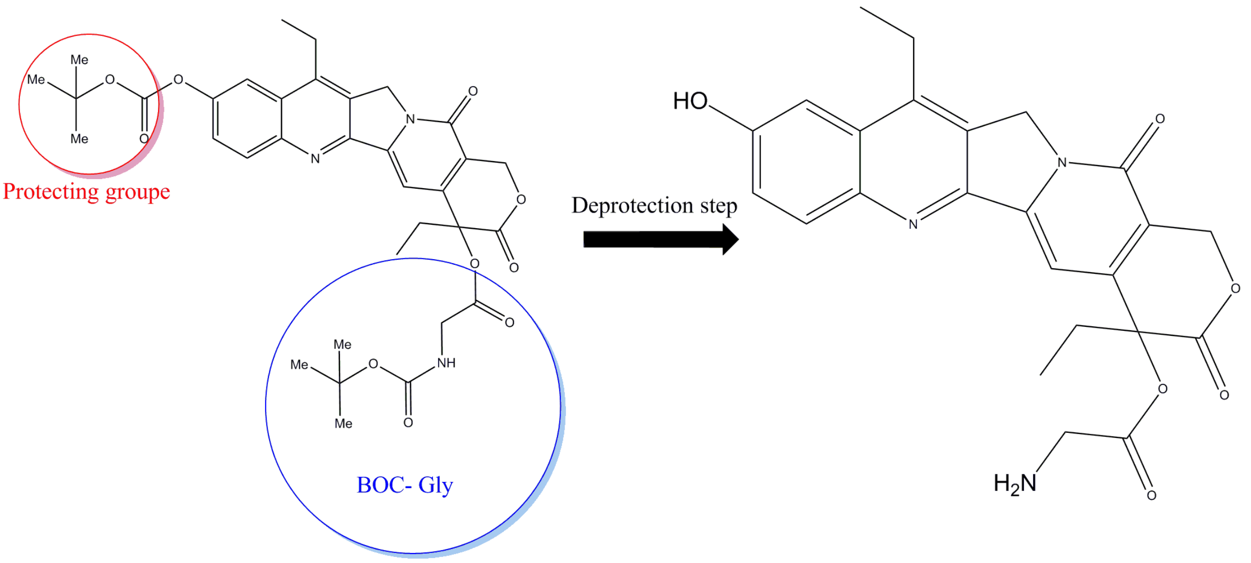

HNMR

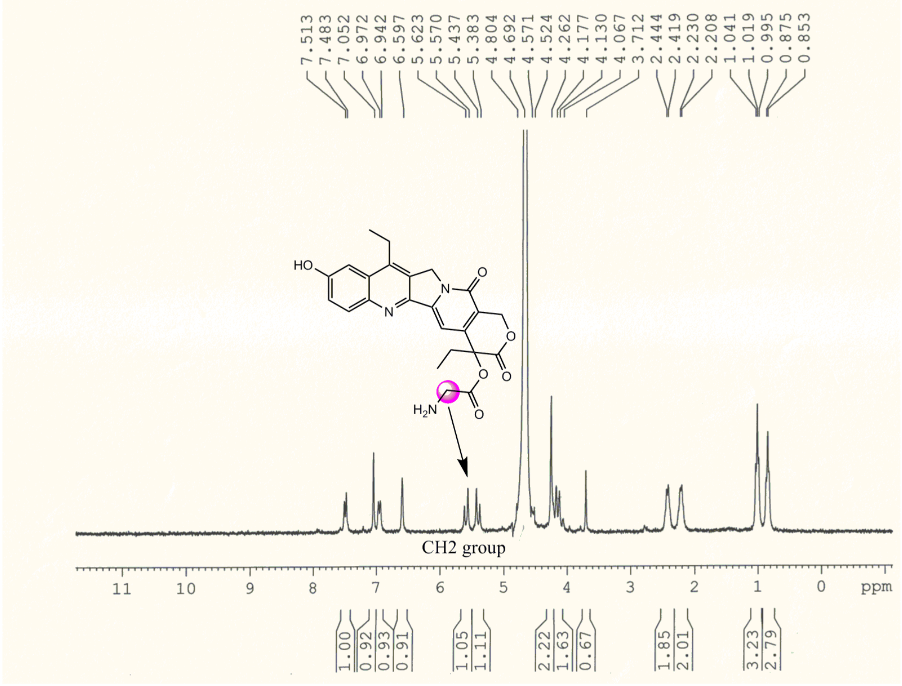

Fig. S3: De-protection of SN38-glycine-BOC to produce SN38–20-*O*-glycine.

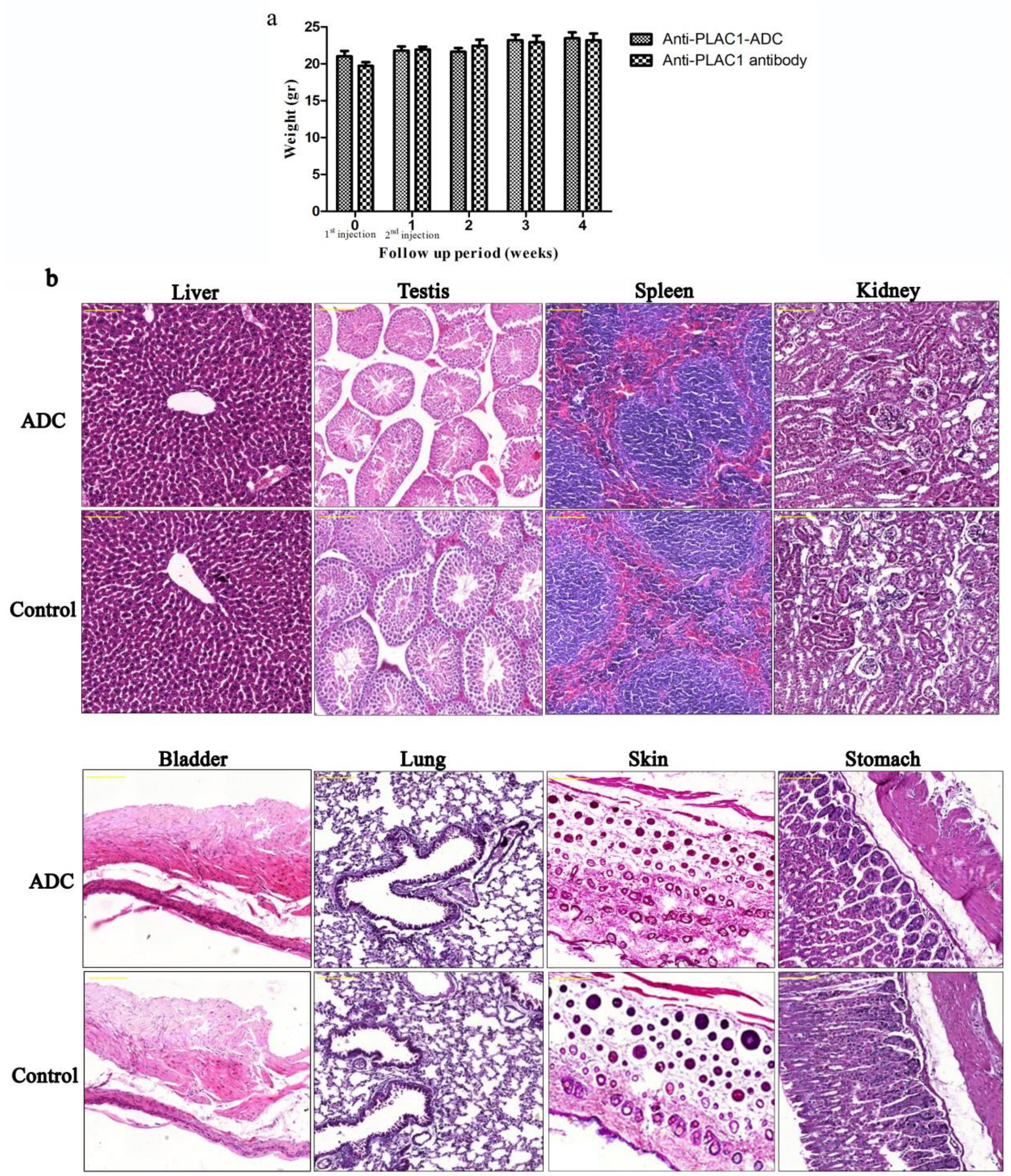

Fig.S4: Histopathological analyses of mouse tissues after administration of anti-PLAC1-ADC. Balb/c mice were injected intraperitoneally twice at a one-week interval with either anti-PLAC1-ADC or unconjugated anti-PLAC1 antibody and monitored for weight loss (a). Two weeks after the last injection, tissues were removed and processed for histological

examinations. Compared to the control mice, experimental mice exhibited no sign of pathological changes in the tissues examined (b). Scale bars: 200 µm.

ATATATCAGACCATCAGAAGGATTTGTATAAAGAGTGACTCTCCTATGAAGGTAAAGGCCACCCCTCTTC
AGTTCCAGTGACTGAGATACATTTTTCCAATCCTGGGGGCAAATACAGACACAGCAAGTTCCTTCTTCCC
TTTGGAAATTTGGCAGCTGCCTTCAC**CAGTGAGCACAAAGCCACATTTC**AAAGGAAACTGACAAATTATC
CCCAGCTGCCAGAAGAAGAAATCCTCACTGGACGGCTTCCTGTTTCCTGTGGTTCATTATCTGATTGGCT
GCAGGGATGAAAGTTTTTAAGTTCATAGGACTGATGATCCTCCTCACCTCTGCGTTTTCAGCCGGTTCAG
GACAAAGTCCAATGACTGTGCTGTGCTCCATAGACTGGTTCATGGTCACAGTGCACCCCTTCATGCTAAA
CAACGATGTGTGTGTACACTTTCATGAACTACACTTGGGCCTGGGTTGCCCCCCAAACCATGTTCAGCCA
CACGCCTACCAGTT**CACCTACCGTGTTACTGAATGTGG**CATCAGGGCCAAAGCTGTCTCTCAGGACATGG
TTATCTACAGCACTGAGATACACTACTCTTCTAAGGGCACGCCATCTAAGTTTGTGATCCCAGTGTCATG
TGCTGCCCCCCAAAAGTCCCCATGGCTCACCAAGCCCTGCTCCATGAGAGTAGCCAGCAAGAGCAGGGCC
ACAGCCCAGAAGGATGAGAAATGCTACGAGGTGTTCAGCTTGTCACAGTCCAGTCAAAGGCCCAACTGCG
ATTGTCCACCTTGTGTCTTCAGTGAAGAAGAGCATACCCAGGTCCCTTGTCACCAAGCAGGGGCTCAGGA
GGCTCAACCTCTGCAGCCATCTCACTTTCTTGATATTTCTGAGGATTGGTCTCTTCACACAGATGATATG
ATTGGGTCCATGTGATCCTCAGGTTTGGGGTCTCCTGAAGATGCTATTTCTAGAATTAGTATATAGTGTA
CAAATGTCTGACAAATAAGTGCTCTTGTGACCCTCATGTGAGCACTTTTGAGAAAGAGAAACCTATAGCA
ACTTCATGAATTAAGCCTTTTTCTATATTTTTATATTCATGTGTAAACAAAAAATAAAATAAAATTCTGA
TCGCAT

Fig.S5: Position of Plac1 PCR primers on NM_0217963. Forward and reveres primers have been highlighted as bold red and blue letters, respectively.

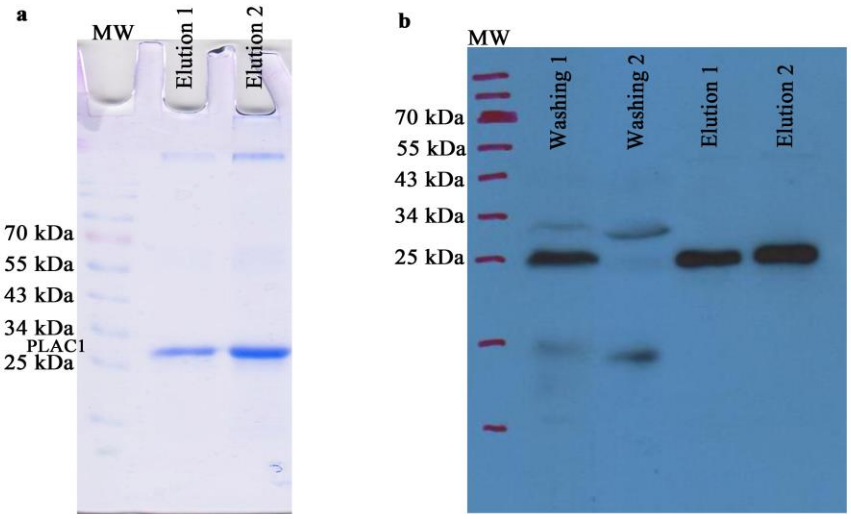

Fig.S6: Characterization of recombinant human PLAC1 (rPLAC1). rhPLAC1 was produced in *E.coli* and purified. The purity of the protein was assessed by SDS-PAGE analysis (a) and Western blot (b) using anti-His tag antibody. In both SDS-PAGE and Western blot analyses, bands of elusion 2 have been presented in the main manuscript.

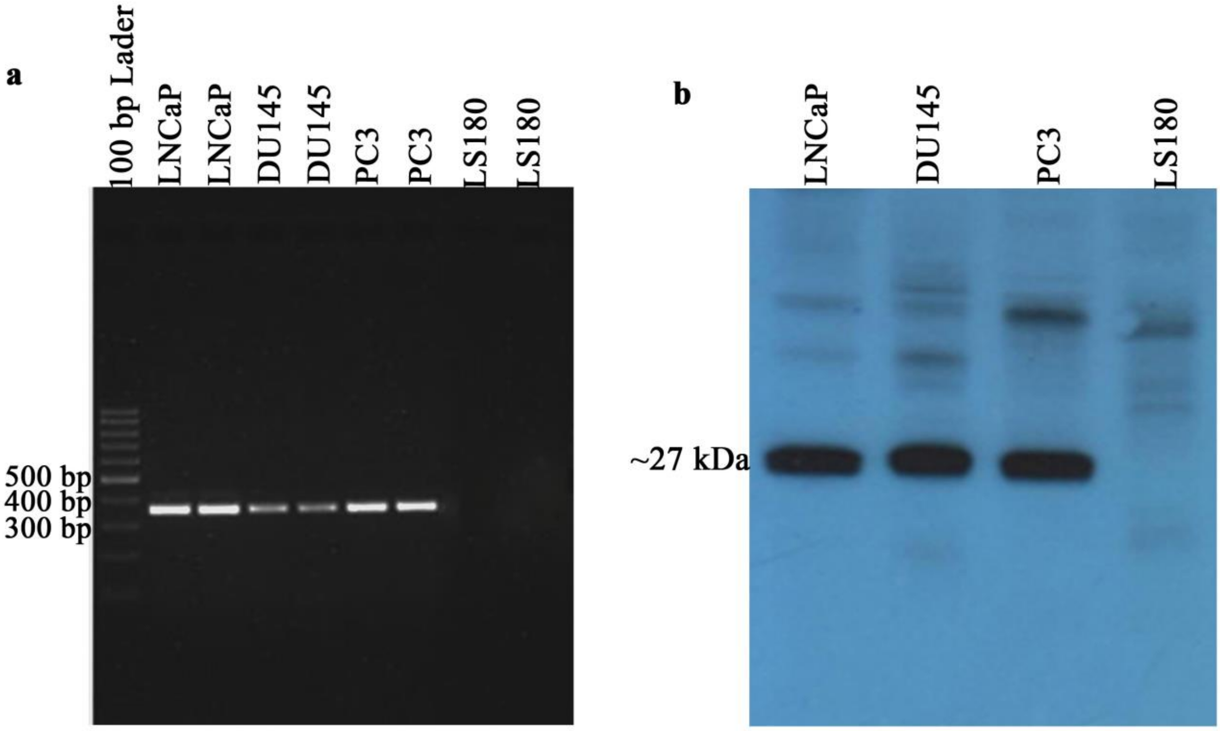

Fig.S7: Characterization of PLAC1 expression by human prostate cancer cell lines. PLAC1 expression in prostate cancer cell lines LNCaP, DU145 and PC3 was assessed by RT-PCR (a) and Western blotting (b).

References:

1. Moon, S.-J. et al. Antibody conjugates of 7-ethyl-10-hydroxycamptothecin (SN-38) for targeted cancer chemotherapy. *Journal of Medicinal Chemistry* **51**, 6916-6926 (2008).
2. Nazari, M. et al. Optimized protocol for soluble prokaryotic expression, purification and structural analysis of human placenta specific-1 (PLAC1). *Protein Expression and Purification* **133**, 139–151 (2017).
3. Ghods, R. et al. High placenta-specific 1/low prostate-specific antigen expression pattern in high-grade prostate adenocarcinoma. *Cancer Immunology, Immunotherapy* **63**, 1319-1327 (2014a).
4. Ghods, R. et al. Immunohistochemical characterization of novel murine monoclonal antibodies against human placenta-specific 1. *Biotechnology and Applied Biochemistry* **61**, 363-369 (2014b).
5. Nejadmoghaddam, M.R., Chamankhah, M., Zarei, S. & Zarnani, A.H. Profiling and quantitative evaluation of three Nickel-Coated magnetic matrices for purification of recombinant proteins: helpful hints for the optimized nanomagnetisable matrix preparation. *Journal of nanobiotechnology* **9**, 31 (2011).
6. Moravej, A. et al. Evaluation of thyroglobulin expression in murine reproductive organs during pregnancy. *American Journal of Reproductive Immunology* **64**, 97-103 (2010).
7. Lawrence, M.G. et al. A preclinical xenograft model of prostate cancer using human tumors. *Nature protocols* **8**, 836 (2013).
8. Sepehri, N. et al. Human Serum Albumin Conjugates of 7-Ethyl-10-hydroxycamptothecin (SN38) for Cancer Treatment. *BioMed Research International* **2014**, 11 (2014).
